# Clinical situations for which 3D printing is considered an appropriate representation or extension of data contained in a medical imaging examination: neurosurgical and otolaryngologic conditions

**DOI:** 10.1186/s41205-023-00192-w

**Published:** 2023-11-27

**Authors:** Arafat Ali, Jonathan M. Morris, Summer J. Decker, Yu-hui Huang, Nicole Wake, Frank J Rybicki, David H Ballard

**Affiliations:** 1 Department of Radiology, Henry Ford Health, Detroit, MI USA; 2 Department of Radiology, Mayo Clinic, Rochester, MN USA; 3 Division of Imaging Research and Applied Anatomy, Department of Radiology, University of South Florida Morsani College of Medicine, Tampa, FL USA; 4 Department of Radiology, University of Minnesota, Minneapolis, MN USA; 5 Department of Research and Scientific Affairs, GE HealthCare, New York, NY USA; 6 Center for Advanced Imaging Innovation and Research, Department of Radiology, NYU Langone Health, New York, NY USA; 7 Department of Radiology, University of Cincinnati College of Medicine, Cincinnati, OH USA; 8 Mallinckrodt Institute of Radiology, Washington University School of Medicine, Saint Louis, MO USA

**Keywords:** 3D printing, Appropriateness, Guidelines, Quality, Radiology, Additive Manufacturing, Anatomic model, Neurology, And Neurosurgery

## Abstract

**Background:**

Medical three dimensional (3D) printing is performed for neurosurgical and otolaryngologic conditions, but without evidence-based guidance on clinical appropriateness. A writing group composed of the Radiological Society of North America (RSNA) Special Interest Group on 3D Printing (SIG) provides appropriateness recommendations for neurologic 3D printing conditions.

**Methods:**

A structured literature search was conducted to identify all relevant articles using 3D printing technology associated with neurologic and otolaryngologic conditions. Each study was vetted by the authors and strength of evidence was assessed according to published guidelines.

**Results:**

Evidence-based recommendations for when 3D printing is appropriate are provided for diseases of the calvaria and skull base, brain tumors and cerebrovascular disease. Recommendations are provided in accordance with strength of evidence of publications corresponding to each neurologic condition combined with expert opinion from members of the 3D printing SIG.

**Conclusions:**

This consensus guidance document, created by the members of the 3D printing SIG, provides a reference for clinical standards of 3D printing for neurologic conditions.

**Supplementary Information:**

The online version contains supplementary material available at 10.1186/s41205-023-00192-w.

## Background

In 2018, the Radiological Society of North America (RSNA) three dimensional (3D) printing Special Interest Group (SIG) published guidelines for medical 3D printing and appropriateness for certain clinical scenarios including congenital heart disease, craniomaxillofacial pathologies, genitourinary pathologies, musculoskeletal pathologies, vascular pathologies, and breast pathologies [1]. Currently, medical 3D printing is performed for neurosurgical and otolaryngologic conditions such as pathology involving the skull base, brain tumors unrelated to the skull base, and craniosynostosis, but without evidence for when 3D printing is appropriate. The purpose of this document is to identify the clinical conditions for neurosurgical and otolaryngologic 3D printing, and then vet, vote and publish recommendations on their appropriateness.

## Methods

The 3D SIG identified clinical situations for 3D printing of neurologic conditions, and then provide recommendations for when 3D printing is considered usually appropriate, maybe appropriate, and rarely appropriate [2]. Strength of evidence was determined by literature review. Consensus among 3D printing SIG members is used when there is a paucity of evidence.

The SIG Guidelines Chairperson managed the ratings of this document via a vote among SIG members. The results of the ratings follow the established 1–9 format (with 9 being the most appropriate):

1–3, red, rarely appropriate: There is a lack of a clear benefit or experience that shows an advantage over usual practice.

4–6, yellow, maybe appropriate: There may be times when there is an advantage, but the data is lacking, or the benefits have not been fully defined.

7–9, green, usually appropriate: Data and experience shows an advantage to 3D printing as a method to represent and/or extend the value of data contained in the medical imaging examination.

Clinical scenarios were organized by pathologies unique to the three main regions of the skull base, extra-axial tumors unrelated to the skull base, the updated 2021 world health organizations (WHO) definitions of intra-axial brain tumors and craniosynostosis [3–5]. A major treatise in neuroimaging served as a guide for search terms (Appendix 1), to ensure an exhaustive search [6]. Afterwards, an English language PubMed literature search and an AUC document structure using standard categories for assessment were created. The supporting evidence was obtained through structured PubMed searches. From each search result the relevant articles written in English were curated by consensus between physicians with expertise in 3D printing and neuroimaging. Publications were deemed ineligible if they solely focused on bioprinting, virtual or augmented reality, were not related to human subjects, or were review articles without new patient data. All final included literature and recommendations of this section were vetted and approved by vote of Special Interest Group members virtually at the November 21, 2022 SIG Appropriateness Committee Meeting. Afterwards, the ratings and associated literature were posted on the SIG’s members-only online forum and comments could be made by SIG members for a 2-week period. All included studies were graded with a strength of evidence assessment, using as a methodology the assignment used by the American College of Radiology [2]. This manuscript represents the findings and conclusion of the 3D printing SIG and does not represent an endorsement by the RSNA.

## Results

Table 1 provides evidence-based [7–161] appropriateness ratings, supplemented by expert opinion when there was a paucity of peer-review data, to define and support the use of 3D printing for patients with neurologic conditions. The citations included in forming the appropriateness recommendations and the strength of evidence assessment are presented in Appendices 1 and 2 respectively.

**Table 1:** Appropriateness Ratings for Neurosurgical and Otolaryngologic Conditions

Clinical Condition	Rating	References
**Skull Base: Anterior**		
Olfactory Groove Meningioma: Simple	**3**	[7–10]
Olfactory Groove Meningioma: Complex	**9**	[7–9]
Tuberculum Sella / Planum Sphenoidale Meningioma: Simple (Class I)	**3**	[7–12]
Tuberculum Sella / Planum Sphenoidale Meningioma: Complex (Class II-III)	**7**	[7–12]
Esthesioneuroblastoma: Kadish Group A-B	**3**	
Esthesioneuroblastoma: Kadish Group C	**8**	
Sinonasal tumors: Simple	**3**	[13, 14]
Sinonasal tumors: Complex	**8**	[13, 14]
Juvenile Nasopharyngeal Angiofibroma (JNA) invading the skull base (stage III)	**5**	
Frontal Sinus Infection invading skull base	**2**	[13, 14]
Frontal /Ethmoid Sinus Mucocele with Intracranial Extension	**5**	[14, 15]
**Skull Base: Middle**		
Pituitary Macroadenoma: Simple (Knosp 1–2 or Hardy 0–3 A-C)	**2**	[9, 16–21]
Pituitary Macroadenoma: Complex (Knosp 3–4 or Hardy 4, D-E)	**9**	[9, 16–21]
Pituitary Carcinoma	**6**	[19–21]
Craniopharyngioma: Adamantinomatous	**8**	[8, 9, 22, 23]
Craniopharyngioma: Papillary	**7**	[8, 9, 22, 23]
Anterior Clinoid Meningiomas: Simple (Al-Mefty Type II, type A)	**1**	[11]
Anterior Clinoid Meningiomas: Complex (Al-Mefty Type I, type B,C)	**6**	[9, 11]
Optic Nerve Sheath meningioma: Simple	**2**	
Optic Nerve Sheath meningioma: Complex (Al-Mefty III)	**6**	
Sphenoid Wing Meningiomas: Simple or Group 1, No Cavernous Sinus involvement	**3**	[8, 10, 24–26]
Sphenoid Wing Meningiomas: Complex or Group II, Cavernous Sinus involvement	**8**	[8–10, 24–28]
Nasopharyngeal Tumor: TNM designation T3 or T4	**5**	
**Skull Base: Posterior**		
Cerebellopontine Angle: Vestibular Schwannoma: Simple (Koos Grade I and II)	**1**	
Cerebellopontine Angle: Vestibular Schwannoma: Complex (Koos Grade III -IV)	**9**	[29]
Cerebellopontine angle tumors, not otherwise specified	**6**	[10, 30]
Petroclival Meningioma:	**7**	[31, 32]
Chordoma	**5**	
Chondrosarcoma	**7**	[33]
Foramen Magnum Meningioma: Simple	**2**	[10]
Foramen Magnum Meningioma: complex	**7**	[10]
Chiari I Malformation	**3**	[34]
**Skull Base: Can occur anywhere**		
Metastasis: Simple	**2**	
Metastasis: Complex	**5**	
Solitary Fibrous Tumor (hemangiopericytoma)	**6**	
Myeloma: Simple	**2**	
Myeloma/Plasmacytoma: Complex	**7**	
Perineural Tumor Spread Intracranially	**1**	
Lymphoma: Simple	**1**	
Lymphoma: Complex	**4**	
Fibrous Dysplasia	**8**	
Hemangioma: Simple	**1**	
Hemangioma: Complex	**7**	
Ameloblastoma	**7**	[35]
Schwannoma	**6**	[12, 36]
Encephalocele	**6**	
Meningioma: NOS Simple	**3**	[10, 37, 38]
Meningioma: NOS Complex	**8**	[10, 37, 38]
**Skull Base: Congenital or Acquired deformity**		
Basilar Invagination, Platybasia, Craniocervical or Craniovertebral anomalies	**8**	[39–46]
**Skull Base: Temporal Bone**		
Inflammatory: Cholesteatoma/Cholesterol Granuloma	**6**	[33, 47–54]
Infection	**1**	[48]
Neoplasm: Primary Temporal Bone	**5**	[48, 52, 53, 55–72]
Dehiscence Semicircular canal	**5**	[64, 73, 74]
CSF Leak	**5**	[75–79]
Cochlear Implant Placement	**3**	[80–83]
Osteoconductive Implant Placement	**7**	[64, 80, 81, 84–90]
**Brain Tumors**		
Intra-axial Glial Neoplasms	**3**	[91–103]
Intra-axial Non-Glial Neoplasms	**3**	
Intraventricular Tumors	**1**	
Brain Stem Neoplasms	**3**	
Tumor of the Pineal Region	**3**	
Extra-axial Neoplasms (not elsewhere specified)	**2**	
Meningiomas not related to Skull Base: Simple	**2**	
Meningiomas not related to Skull Base: Complex	**7**	
CNS Lymphoma	**2**	
Lesions affected the cranial nerves (not elsewhere specified)	**5**	[17, 32, 104]
**Craniosynostosis**		
Simple Single Suture: Open Repair	**7**	[105–110]
Simple Single Suture: Endoscopic repair	**7**	[105–110]
Complex Multiple Suture: Open repair	**8**	[105–110, 26]
Complex Syndromic	**8**	
Metopic bandeau	**8**	
**Cerebrovascular Disease**		
Cerebral Aneurysms	**7**	[111–145]
Cerebral venous and Dural venous sinus disease	**6**	[146, 147]
Arteriovenous malformations	**7**	[148–153]
Vascular simulation (hemodynamics and interventions)	**9**	[121, 141, 144, 151, 154–157]
Vessel injury	**2**	[158]
Atherosclerotic disease	**2**	[159]
Ischemic and hemorrhagic brain injury	**2**	[160, 161]

## Discussion

### Skull base

The skull base is a complex anatomic region that separates the intracranial tissues from the extracranial compartments with multiple neural and vascular structures extending through foramina and fissures. Lesions in this region may originate within the skull base itself, from intracranial tissues and extend inferior, or from extracranial soft tissues extending superiorly [162–164]. Given the complexity of pathology in this region there is no one classification for neoplastic and non-neoplastic pathologies. Pathologies of the skull base are typically subdivided both clinically and radiographically by anatomic region: the anterior, middle, and posterior skull base.

### Anterior skull base

The anterior skull base separates the intracranial content from the nasal cavity. There are many histologic tissue types are present in the anterior skull base. Primary tumors of this area may be derived from the bone, paranasal sinuses, nasopharynx, dura, cranial nerves, pituitary gland and brain.

#### Olfactory groove meningiomas

The olfactory groove is a paired depression in the cribriform plate on either side of the crista galli. It transmits the olfactory nerves and anteriorly contains a small foramen for the nasociliary nerve, a branch of the ophthalmic nerve. Olfactory groove meningiomas account for approximately 10% of intracranial meningiomas. Because of their slow growth and anatomic location, patient with olfactory groove meningiomas typically present later in the natural history of the disease with larger size of tumors, approximately 15% of which extend into the nasal cavity [164, 165]. Multiple surgical approaches to remove these tumors are used including bifrontal, unilateral frontal, and pterion craniotomies. Endoscopic approaches with the aid of an otorhinolaryngologist are also described [166]. No formal classification system exists for olfactory groove exists. Therefore, a binary distinction of simple and complex olfactory groove meningioma is used in this report. Simple olfactory groove meningiomas are defined as well circumscribed tumors measuring less than 4 cm without significant hyperostosis, extension into the nasal cavity, brain invasion, significant brain edema, or encasement of major vascular structures. Complex olfactory groove meningiomas are categorized as those measuring greater than 4 cm with irregular margins, significant hyperostosis of the adjacent bone, greater than 25% calcification, brain invasion, significant brain edema, encasement of the anterior communicating artery or anterior cerebral artery branches, and/or extension into the nasal cavity. 3D Printing case series and case reports have shown benefit in preoperative planning, patient informed consent, intraoperative guidance, shortening operative time, and improving anatomic understanding during surgical removal [7–9].

#### Tuberculum sella meningiomas

Meningiomas of the tuberculum sella arise from the limbus sphenoidale, chiasmatic sulcus, and tuberculum. They comprise approximately 3–10% of all intracranial meningiomas and typically present earlier than olfactory groove meningiomas due compression of the optic chiasm leading to visual symptoms [167, 168]. Tuberculum sellae meningiomas characteristically lie in a suprasellar sub-chiasmal midline position resulting in posterior and superior displacement of the optic chiasm and lateral displacement of the pre-chiasmatic optic nerve. Management ideally consists of gross-total resection without injury to neighboring vital structures. Surgical approaches included extended bifrontal, unilateral frontal, pterional, and fronto-temporo-orbito-zygomatic (FTOZ) trajectories [169]. Palani et al. proposed a scoring system for classification which factors tumor size, optic canal invasion, vascular encasement of the internal cerebral and anterior cerebral arteries, brain invasion, previous surgery, or previous radiation [169]. Class I (0–3 points), class II (4–7 points), and class III (8–11 points) disease have prognostic implication of surgical risk, intraoperative vascular injury, subtotal resection, need for adjuvant radiation, and likelihood of visual symptom improvement [170]. 3D printing is beneficial in more complex tumors (class II-III) of this region for improved preoperative anatomic understanding, intraoperative guidance, improved patient informed consent and trainee education.

#### Olfactory neuroblastoma (Esthesioneuroblastoma)

Olfactory neuroblastoma, also referred to as esthesioneuroblastoma, is a rare malignant tumor of neuroectodermal origin thought to arise from the olfactory epithelium [171]. There is bimodal age distribution with one peak in young adult patients (approximately 2nd decade of life) and a second peak in the 5th to 6th decades [172]. These tumors are most frequently staged using a system proposed by Kadish et al. in 1976 which includes group A: limited to the nasal cavity, group B: limited to the nasal cavity and paranasal sinuses, and group C: extended beyond the nasal cavity and paranasal sinuses into the skull base, intracranial compartment, or orbits. Distant metastatic disease also qualifies group C disease [173]. An additional group was added by Chao et al. in 2001 including group D: cervical nodal metastases. Treatment usually involves combinations of chemotherapy, radiotherapy and surgical excision [174]. Prognosis is significantly affected by the presence of distant metastases. No specific literature exists related to benefits of 3D printing for esthesioneuroblastoma; however, it has been shown by members of the 3D printing SIG to successfully demonstrate relationships of tumor to critical intracranial anatomy and vascular structures in Kadish group C tumors. Complex trans-osseous tumors with vascular encasement and displacement of neural structures stand to benefit the most from preoperative planning and patient specific 3D Printing.

#### Sinonasal tumors

Sinonasal tumors are a heterogenous group of tumors that originate in the sinus or nasal cavity, of which squamous cell carcinomas are the most common (80%) [175]. Adenoid cystic carcinoma is the second most common and most likely to recur after surgery (75–90%) [176, 177]. Perineural tumor spread is the hallmark of adenoid cystic tumors which sometimes presents with late recurrences. Adenocarcinoma represents 10% of nasal cavity tumors. Other rarer tumors include mucoepidermoid, sinonasal melanoma, and sinonasal undifferentiated carcinoma which is the most aggressive [178]. No peer reviewed literature related to 3D printing exists for this subgroup presently; however, members of the 3D SIG have 3D printed patient specific aggressive sinonasal tumors extending intracranially for preoperative planning and found it beneficial.

#### Juvenile nasopharyngeal angiofibroma (JNA)

Juvenile nasopharyngeal angiofibroma (JNA) is a benign but locally aggressively vascular tumor that may involve the anterior skull base and extend intracranially. Patients are typically young males who present with epistaxis or chronic otomastoiditis due to obstruction of the Eustachian tube. The staging system proposed by Sessions et al. is the most commonly used and divides tumors into three stages with extension into the skull base qualifying stage III disease [179]. There is no peer reviewed literature related to 3D printing and JNA.

#### Frontal sinus infection

Frontal sinus infection can be complicated by intracranial extension if left untreated or in immunocompromise patients. Intracranial complications include the formation of brain abscesses, subdural empyema, meningitis, cavernous sinus thrombosis, or osteomyelitis [180, 181]. While most infectious etiologies do not merit a 3D printed model, Jung et al. published a case report where 3D printing was used for reconstruction of the frontal bone after severe infection of the frontal sinuses [182].

#### Frontal sinus mucocele

A mucocele of the paranasal sinus is an accumulation of mucoid secretion and desquamated epithelium within the sinus resulting in benign cyst-like expansion of the sinus walls. Approximately 60–89% occur in the frontal sinus, followed by 8–30% in the ethmoid sinuses, and less than 5% in the maxillary sinus [183]. The treatment of mucoceles is surgical to drain the mucocele and ventilate the sinus and prevent recurrences [184]. Sanchez-Gomez published a case series of 7 patients where 3D printing using stereolithography was used to improve preoperative planning, patient specific anatomic understanding, and reducing intraoperative time [15].

### Middle skull base

The central skull base represents the junction between the intracranial contents, the bone of the skull base, the orbits, the paranasal sinuses, and the suprahyoid neck. It contains the anterior clinoid processes, sphenoid wings, sella, cavernous sinus.

#### Pituitary macroadenoma

Pituitary adenomas are relatively common tumors arising from adenohypophyseal cells and account for 10–15% of all intracranial neoplasms [185]. Pituitary adenomas have been classified according to the clinical, radiological, and endocrinological findings, tumor size, and invasion of adjacent structures. Pituitary adenomas are divided into microadenomas and macroadenomas by a cutoff size of 10 mm. Pituitary macroadenomas (greater than 1 cm) often extend into the suprasellar compartment giving rise to a classic “snowman” or “Figure of 8” morphology. Invade the cavernous sinus occurs in 6–10% of cases, limiting surgical resectability [186, 187]. There are 3 main classifications of Pituitary Adenomas; Hardys classification which incorporates bone invasion inferiorly into the sphenoid sinus (grade 0–4) and suprasellar involvement (grade A-E) and Knosp classification of cavernous sinus invasion (Grade I-IV) [188–190]. 3 case series, 1 case report, and 1 randomized control trial of 20 patients demonstrated improved preoperative planning, intraoperative guidance, patient education, blood loss and operative times [16–18].

#### Craniopharyngioma

Craniopharyngiomas are midline suprasellar tumors which are relatively benign (WHO grade I), but locally aggressive. They originate from epithelial remnants of Rathke’s pouch and are a formidable neurosurgical resection as they are intimately associated with the hypothalamus and optic apparatus. They are classified by location as retrochiasmatic, prechiasmatic, intraventricular (third ventricle), and intrasellar. Several surgical approaches have been created depending on the age of the patient, location, and size of the tumor [191]. Pathologically there are two subtypes, papillary (PCP) and adamantinomatous (ACP). ACPs are more common in children are composed of cystic “motor oil-like” components as well as solid components with frequent calcification. In contrast, PCPs are more common in adults, rarely calcified, mostly solid, and well-circumscribed with clear cyst contents [192]. Advancements in imaging have led to several described classification schemes, but no single scheme is widely used [191, 193–196]. Surgical goals must be balanced with the potential morbidity of hypothalamic or optic apparatus injury; hence, preoperative understanding of tumor anatomy is crucial. Guo published a case series of 355 craniopharyngiomas, 45 of which had 3D printed models used for preoperative planning [22]. This study demonstrated improved preoperative anatomic understanding which aided in choosing surgical approach. Other smaller case series demonstrate similar findings [8, 9].

#### Anterior clinoid meningiomas

Anterior clinoidal meningiomas arise from the meningeal covering of the anterior clinoid process. These meningiomas are distinct from the more commonly discussed sphenoid wing meningiomas with unique anatomic landmarks, surgical outcomes, and clinical experience [197]. They are divided further into 3 subcategories based on their relation to the anterior clinoidal process and ease of resection.

*Type I* - Clinoidal meningiomas originate from the inferomedial surface of the clinoidal process proximal to the distal carotid ring.

*Type II* - Clinoidal meningiomas originate from the superolateral surface of the clinoid process, leading to widening of the sylvian fissure.

*Type III* – Clinoidal meningiomas originate at the optic foramen and extend into the optic canal.

Preoperative imaging has suboptimal sensitivity for detection of tumor involving the clinoid process (approximately 75%) and the clinoid process is typically removed [198]. Limited literature exist surrounding the utilization of 3D printing in preoperative planning for these meningiomas [9, 11]. However, members of the 3D printing SIG have printed patient specific models in this region for preoperative planning. Given the close association with the cavernous sinus and intracranial internal carotid artery, 3D printing maybe appropriate for this disease process.

#### Optic nerve sheath meningioma

Optic nerve sheath meningiomas are rare benign neoplasms originating in the arachnoid cap cells of the meninges surrounding the optic nerve. While benign, they are a significant source of morbidity due to loss of vision, disfigurement from proptosis or potential operative morbidity. While these tumors are rare, they account for one-third of meningiomas involving the orbit [199]. Surgical resection carries the risk of blindness, creating the need to balancing growth of the tumor vs. potential morbidity from resection. These tumors are typically unilateral except in the context of neurofibromatosis type 2 [200]. These are slow growing tumors, therefore surgical management is only considered in situations where tissue diagnosis is required, tumor demonstrates progressive posterior extension into the intracranial compartment, complete vision loss is pre-existing and en bloc resection is possible, or in patients who have significant orbital disfigurement [201, 202]. There is no peer reviewed literature related to 3D printing and optic nerve sheath meningioma. Members of the 3D SIG have created models for Al-Mefty category III tumors and found it to be useful for preoperative planning and intraoperative guidance [197].

#### Sphenoid wing meningioma

Sphenoid wing meningiomas account for 11–20% of intracranial meningiomas. The location of the tumor has been further divided into 3 groups: (1) medial; (2) middle; and (3) lateral. En plaque meningiomas occur in this location commonly and are characterized by sheetlike dural thickening and bone hyperostosis. Management of meningiomas in this location can be difficult, especially medially, due to proximity of neurovascular structures traversing the adjacent neural foramina and the adjacent cavernous sinus contents. There are varied surgical approaches beyond the pterion craniotomy, therefore preoperative localization of the anatomic extension of the tumor is important [203, 204]. Extent of resection and morbidity can depend on cavernous sinus involvement, encasement of the anterior cerebral or middle cerebral arteries, orbital apex involvement, and bony hyperostosis [27, 205–207]. 3D printed models have been used in several case series which demonstrated improved preoperative planning, selection of surgical approach, anatomic understanding of critical neurovascular structures in relationship to tumor, and patient education [8, 9, 24, 25, 28].

#### Nasopharyngeal carcinoma

Nasopharyngeal carcinoma is the most common tumor of the nasopharynx for which radiation and chemotherapy are the primary modalities for therapy [208]. Members of the 3D SIG anecdotally report clinical utility for tumors with involvement of adjacent bony structures or those with intracranial extension, TNM designations T3 and T4 respectively [209]. There is no peer reviewed literature describing the use of 3D printing for nasopharyngeal carcinoma.

### Posterior skull base

The clivus forms the anterior aspect of the posterior skull base and extends inferiorly to the foramen magnum. Laterally the posterior skull base is formed by the posterior surface of the petrous portion of the temporal bone and the mastoid portion of the temporal bone. Detailed knowledge of the foramen and the neurovascular structures traversing them is essential in surgical management of tumors in this location.

#### Meningiomas

Posterior cranial fossa meningiomas account for approximately 8–10% of all intracranial meningiomas [6]. There are few published reports describing the benefit of a 3D printed model for resection of posterior fossa meningiomas in the petroclival region. As our ability to visualize and accurately segment skull base structures improve, we anticipate that the need and utility of such models will increase.

#### Vestibular schwannoma

Vestibular schwannoma, also known as acoustic schwannomas or acoustic neuromas, are benign tumors which comprise the vast majority of cerebellopontine angle masses (~ 85–90%) [6]. There are few published reports for the use of 3D printing for vestibular schwannomas [17]. However, we have anecdotally noted a high demand for these models at our institution for presurgical planning and surgical trainee education. Specifically, these models have been used to determine the surgical approach and proximity of the tumor with cranial nerve VII.

#### Chordomas

Chordomas are a locally aggressive primary malignant neoplasm which occur at the midline, arising at any point along the course of the primitive notochord. Spheno-occipital chordomas, also known as clival chordomas, are located intracranially at the midline and are less common compared to sacral or spinal chordomas. Endoscopic and multiple open surgical approaches are described in the management of clival chordomas [210]. There are no published reports for 3D printing for planning of surgical resection of clival chordomas. The need for extensive drilling of bony structures in the skull base during the surgical resection of these tumors suggests preoperative visualization and simulation with a patient specific 3D printed model could add value.

#### Petrous apex

The petrous apex is a pyramidal shaped bone of the middle skull base which is formed by the medial aspect of the temporal bone. Diseases affecting the temporal bone are wide ranging, many of which are amenable to open or endoscopic surgical techniques. Few reports currently exist for lesions of the petrous apex including cases of chondrosarcoma, cholesteatoma, and a petrous apex cyst [33, 47, 50]. All reports reaffirm that 3D printed models of petrous apex pathologies are accurate, aid in preoperative planning and improve patient safety.

### Temporal bone

Anatomy of the temporal bone is complex and surgical procedures involving the temporal bone are technically challenging. The ability to plan and rehearse procedures on a life-like model are invaluable to the field of otologic surgery. A plethora of reports demonstrate the feasibility of temporal bone models produced by 3D printing technology to be both accurate, cheap and reproducible.^55,70 48,50–53,58−69,71,72,74^ 3D printed temporal bone models have proven both qualitatively and quantitatively accurate compared to temporal bone anatomy visualized on imaging as well cadaveric specimens [48, 52, 72, 74].

#### Ossicular chain

The ossicular chain is comprised of the malleus, incus and stapes bones within the middle ear cavity. Size compatible 3D printed biocompatible materials for use as prosthetics have been shown possible in cadaveric models [85, 90]. However, this application for 3D printing has not been proven in case reports or randomized controlled trials.

#### Labyrinth

One of the emerging indications for patient specific models of the temporal bone is pre-operative planning for cochlear hearing device implantation. Minute structures of the inner ear, although challenging to segment and print in true size, have been demonstrated to be feasible in multiple studies [81, 84, 86, 87]. Patient specific models for this indication have been shown to reduce operative time, reduce overall cost, increase surgical precision and reduce complication in a small case series for implantation of a cochlear hearing device [82]. Authors have used both 3D visualization software and 3D printed models for volumetric studies for various types of inner ear pathologies such as incomplete partition and enlarged vestibular aqueduct syndromes [88]. In a single study, authors suggest the feasibility for creating custom implants for the indication of superior semicircular canal dehiscence [89].

#### Cholesteatoma

Cholesteatomas are an overgrowth of epithelial cells occurring the middle ear cavity and temporal bone which occasionally require surgical removal. CT imaging is the primary preoperative imaging tool guiding preoperative planning. The addition of a 3D printed model for surgical planning has been shown accurate in reproducing anatomy, particularly for patients with complex anatomy [54].

### Congenital or acquired deformity of the skull base

Basilar invagination, platybasia and other craniovertebral anomalies, congenital or acquired, can be challenging to manage operatively. Multiple case reports and case series demonstrate that rehearsal of individualized skull reconstruction with an anatomic model has been shown to improve surgeon confidence, reduce operative risk and improve outcomes [26, 39–46].

### Brain tumors

The global incidence of primary malignant brain tumors in adults is approximately 3.7 per 100,000 for males and 2.6 per 100,000 for females, with even higher rates in developed countries [211]. 3D printing technology has been used in preoperative planning for tumor resections and for radiosurgical guides.^91–100 101,102,151^ Unlike resection of tumors elsewhere in the body, outcomes from brain tumor resection are heavily surgical performance based. Literature supporting improved performance with 3D printed models used for preoperative planning and simulation justify their cost [91–103].

### Cerebrovascular disease

3D printed vascular models are often limited to treatment of complex intracranial vascular pathologies for clinical decision making. However, there is a large body of evidence reporting the use of 3D printed vascular models, both simple and complex, for education and surgical simulation.^111–153,158−161^ Few authors have utilized 3D printing for pre-surgical planning of arteriovenous malformation resection [150, 153]. Models of dural venous sinuses and cerebral venous anatomy are not well reported in the literature; however, fabrication of these models are feasible.

## Conclusion

This document provides clinical appropriateness for 3D printing for patients with neurosurgical and otolaryngologic conditions. Adoption of common clinical standards regarding appropriate use, information and material management, and quality control are needed to ensure the greatest possible clinical benefit from 3D printing. With accruing evidence for utility and value in 3D printing, it is anticipated that this consensus document, created by the members of the 3D printing Special Interest Group, will provide information that can be used for future clinical standards of 3D printing.

### Electronic supplementary material

Below is the link to the electronic supplementary material.

Supplementary Material 1

Supplementary Material 2

## Data Availability

The datasets used and/or analyzed during the current study are available from the corresponding author on reasonable request.

## List of abbreviations

RSNA: Radiological Society of North America
SIG: Special Interest Group
