# Supplementary material for: Clinical situations for which 3D printing is considered an appropriate representation or extension of data contained in a medical imaging examination: neurosurgical and otolaryngologic conditions
Source: 3D Print Med. 2023 Nov 27;9:33. doi: 10.1186/s41205-023-00192-w (PMC10680204; doi:10.1186/s41205-023-00192-w)
Supplement: Supplementary file 1 — Supplementary Material 1 [file 41205_2023_192_MOESM1_ESM.docx]

Appendix 1. Structured PubMed Search terms

1. Skull Base
   - 1. General Search terms – ((3D Printing) AND (Skull Base)) OR ((Rapid Prototyping) AND (Skull Base))
   1. Pituitary
      1. ((3D Printing) AND (Pituitary)) OR ((Rapid Prototyping) AND (Pituitary)) OR ((3D Printing) AND (Macroadenoma)) OR ((rapid prototyping) AND (Pituitary)) OR ((3D Printing) AND (transphenoidal)) OR ((rapid prototyping) AND (transphenoidal))
   2. Meningioma
      1. ((3D Printing) AND (Meningioma)) OR ((rapid prototyping) AND (Meningioma))
   3. Craniopharyngioma
      1. ((3D Printing) AND (craniopharyngioma)) OR ((rapid prototyping) AND (craniopharyngioma))
   4. Acoustic Neuroma/vestibular schwannoma
      1. ((3D Printing) AND (acoustic neuroma)) OR ((rapid prototyping) AND (acoustic neuroma)) OR ((3D Printing) AND (schwannoma)) OR ((rapid prototyping) AND (schwannoma))
   5. Chordoma
      1. ((3D Printing) AND (Chordoma)) OR ((rapid prototyping) AND (Chordoma))
   6. Chondrosarcoma
      1. ((3D Printing) AND (Chondrosarcoma)) OR ((rapid prototyping) AND (Chondrosarcoma))
   7. Chiari Malformation
      1. ((3D Printing) AND (Chiari)) OR ((rapid prototyping) AND (Chiari)) OR ((Three Dimensional Printing) AND (Chiari))
   8. Esthesioneuroblastoma
      1. ((3D Printing) AND (esthesioneuroblastoma)) OR ((rapid prototyping) AND (esthesioneuroblastoma))
   9. Basilar Invagination
      1. May be Acquired, Congenital, post traumatic, post infectious (TB), syndromic, inflammatory (RA)
      2. ((3D Printing) AND (basilar invagination)) OR ((rapid prototyping) AND (basilar invagination))
   10. Craniocervical and Craniovertebral junction
       1. ((3D Printing) AND (craniocervical)) OR ((rapid prototyping) AND (craniocervical)) OR ((3D Printing) AND (craniovertebral)) OR ((rapid prototyping) AND (craniovertebral)) AND (Basilar Invagination)) OR ((rapid prototyping) AND (Basilar Invagination)) AND (Platybasia)) OR ((rapid prototyping) AND (Platybasia)) AND (Platybasia)) OR ((rapid prototyping) AND (Platybasia))
   11. CSF leak or Encephalocele
       1. ((3D Printing) AND (CSF)) OR ((rapid prototyping) AND (CSF)) OR ((3D Printing) AND (Encephalocele)) OR ((rapid prototyping) AND (Encephalocele))
2. Temporal Bone
   - 1. General Search terms - ((3D Printing) AND (temporal Bone)) OR ((rapid prototyping) AND (temporal Bone))
   1. Cochlea
      1. ((3D Printing) AND (Cochlea)) OR ((rapid prototyping) AND (Cochlea))
   2. Labyrinth
      1. ((3D Printing) AND (labyrinth)) OR ((rapid prototyping) AND (labyrinth))
   3. Petrous Apex
      1. ((3D Printing) AND (petrous apex)) OR ((rapid prototyping) AND (petrous apex))
   4. Cholesteatoma
      1. ((3D Printing) AND (cholesteatoma)) OR ((rapid prototyping) AND (cholesteatoma))
   5. Paraganglioma
      1. ((3D Printing) AND (paraganglioma)) OR ((rapid prototyping) AND (paraganglioma))
3. Primary and metastatic intraxial tumors
   - 1. General Search terms - ((3D Printing) AND (brain tumor)) OR ((rapid prototyping) AND (brain tumor)) OR ((3D Printing) AND (brain neoplasm)) OR ((rapid prototyping) AND (brain neoplasm)) OR ((3D Printing) AND (brain cancer)) OR ((rapid prototyping) AND (brain cancer)) OR ((3D Printing) AND (CNS tumor)) OR ((rapid prototyping) AND (CNS tumor)) OR ((3D Printing) AND (CNS neoplasm)) OR ((rapid prototyping) AND (CNS neoplasm)) OR ((3D Printing) AND (CNS cancer)) OR ((rapid prototyping) AND (CNS cancer)) OR ((3D Printing) AND (intraaxial)) OR ((rapid prototyping) AND (intraaxial)) OR ((3D Printing) AND (extraaxial)) OR ((rapid prototyping) AND (extraaxial))
   1. Astrocytomas
      1. ((3D Printing) AND (Astrocytoma)) OR ((rapid prototyping) AND (astrocytoma))
   2. Glial Tumors
      1. ((3D Printing) AND (glioma)) OR ((rapid prototyping) AND (glioma))
   3. Neuronal and glioneuronal tumors
      1. ((3D Printing) AND (neuronal tumor)) OR ((rapid prototyping) AND (neuronal tumor))
   4. Pineal gland and germ cell tumors
      1. ((3D Printing) AND (pineal)) OR ((rapid prototyping) AND (pineal)) OR ((3D Printing) AND (germ cell)) OR ((rapid prototyping) AND (germ cell))
   5. Embryonal neoplasia
      1. ((3D Printing) AND (medulloblastoma)) OR ((rapid prototyping) AND (medulloblastoma)) OR ((3D Printing) AND (atypical teratoid rhabdoid tumor)) OR ((rapid prototyping) AND (atypical teratoid rhabdoid tumor)) OR ((3D Printing) AND (ATRT)) OR ((rapid prototyping) AND (ATRT)) OR ((3D Printing) AND (CNS primitive neuroectodermal tumor)) OR ((rapid prototyping) AND (CNS primitive neuroectodermal tumor)) OR ((3D Printing) AND (PNET)) OR ((rapid prototyping) AND (PNET)) OR ((3D Printing) AND (ependymoblastoma)) OR ((rapid prototyping) AND (ependymoblastoma)) OR ((3D Printing) AND (medulloepithelioma)) OR ((rapid prototyping) AND (medulloepithelioma)) OR ((3D Printing) AND (CNS neuroblastoma)) OR ((rapid prototyping) AND (CNS neuroblastoma)) OR ((3D Printing) AND (CNS ganglioneuroblastoma)) OR ((rapid prototyping) AND (CNS ganglioneuroblastoma)) OR ((3D Printing) AND (CNS embryonal tumor)) OR ((rapid prototyping) AND (CNS embryonal tumor))
   6. Meningeal tumors
      1. ((3D Printing) AND (meninges)) OR ((rapid prototyping) AND (meninges)) OR ((3D Printing) AND (meningeal carcinomatosis)) OR ((rapid prototyping) AND (meningeal carcinomatosis)) OR ((3D Printing) AND (dura)) OR ((rapid prototyping) AND (dura))
      2. Meningioma (non-skull base) - ((3D Printing) AND (meningioma)) OR ((rapid prototyping) AND (meningioma))
      3. Hemangiopericytoma - ((3D Printing) AND (Hemangiopericytoma)) OR ((rapid prototyping) AND (Hemangiopericytoma))
      4. Solitary fibrous tumor of the dura - ((3D Printing) AND (solitary fibrous dura)) OR ((rapid prototyping) AND (solitary fibrous dura))
      5. Malt lymphoma - ((3D Printing) AND (CNS MALT lymphoma)) OR ((rapid prototyping) AND (CNS MALT lymphoma))
   7. Cranial nerves, nerve sheath tumors
      1. ((3D Printing) AND (cranial nerves)) OR ((rapid prototyping) AND (cranial nerves)) OR ((3D Printing) AND (nerve sheath)) OR ((rapid prototyping) AND (nerve sheath))
   8. Lymphoma
      1. ((3D Printing) AND (CNS lymphoma)) OR ((rapid prototyping) AND (CNS lymphoma))
   9. Cystic Lesions
      1. ((3D Printing) AND (CNS cyst)) OR ((rapid prototyping) AND (CNS cyst)) OR ((3D Printing) AND (intracranial cyst)) OR ((rapid prototyping) AND (intracranial cyst))
4. Skull
   1. Skull
      1. ((3D Printing) AND (cranioplasty)) OR ((rapid prototyping) AND (cranioplasty))
   2. Craniosynostosis
      1. ((3D Printing) AND (Craniosynostosis)) OR ((rapid prototyping) AND (Craniosynostosis))
5. Cerebrovascular Disease
   1. Terms – Cerebrovascular disease, cerebral arteries, cerebral veins, cerebral aneurysm, stroke, cerebral vascular
   2. Search input - ((3D printing) AND (cerebrovascular disease)) OR ((rapid prototyping) AND (cerebrovascular disease)) OR ((3D printing) AND (cerebral arteries)) OR ((rapid prototyping) AND (cerebral arteries)) OR ((3D printing) AND (cerebral veins)) OR ((rapid prototyping) AND (cerebral veins)) OR ((3D printing) AND (cerebral aneurysm)) OR ((rapid prototyping) AND (cerebral aneurysm)) OR ((3D printing) AND (stroke)) OR ((rapid prototyping) AND (stroke)) OR ((3D printing) AND (cerebral vascular)) OR ((rapid prototyping) AND (cerebral vascular))
6. Epilepsy
   1. ((3D Printing) AND (epilepsy)) OR ((rapid prototyping) AND (epilepsy))
7. Infection
   - 1. ((3D Printing) AND (CNS infection)) OR ((rapid prototyping) AND (CNS infection))
8. Neurodegenerative Disease
   - 1. ((3D Printing) AND (neurodegenerative disease)) OR ((rapid prototyping) AND (neurodegenerative disease)) or ((3D Printing) AND (alzheimers)) OR ((rapid prototyping) AND (alzheimers))
