# Supplementary material for: Clinical situations for which 3D printing is considered an appropriate representation or extension of data contained in a medical imaging examination: neurosurgical and otolaryngologic conditions
Source: 3D Print Med. 2023 Nov 27;9:33. doi: 10.1186/s41205-023-00192-w (PMC10680204; doi:10.1186/s41205-023-00192-w)
Supplement: Supplementary file 2 — Supplementary Material 2 [file 41205_2023_192_MOESM2_ESM.docx]

**Appendix 2**. Grading of each included study with a strength of evidence assessment according to ACR Appropriateness Criteria Evidence Document.^2^ Studies were categorized as either primarily diagnostic (Dx), therapeutic (Tx), or both (Dx and Tx) along with a designation of observational, experimental, or review/other category. The review/other category is designated for studies that did not meet the definitions the ACR Evidence Document^2^ for observational or experimental studies.

| **Reference Number** | **Reference** | **Study Type** | **Patients/Events** | **Study Objective (Purpose of Study)** | **Study Results** | **Study Quality** |
| --- | --- | --- | --- | --- | --- | --- |
| 7 | Zhang H, Liu G, Tong XG, Hang W. Application of three-dimensional printing technology in the surgical treatment of nasal skull base tumor. Zhonghua Er Bi Yan Hou Tou Jing Wai K. 2018;53(10):780-4. doi:10.3760/cma.j.issn.1673-0860.2018.10.012. | Review/Other-Tx | 7 | To explore the application value of three dimensional (3D) printing technique in the surgical treatment of nasal skull base tumor. | Virtual models of the nasal skull base of 7 patients were reconstructed successfully and 3D 1∶1 entity models were produced. The models demonstrated the relationship among the skull, tumors, and adjacent blood vessels. Seven cases were successfully performed. | 4 |
| 8 | D'Urso PS, Barker TM, Earwaker WJ, Bruce LJ, Atkinson RL, Lanigan MW et al. Stereolithographic biomodelling in cranio-maxillofacial surgery: a prospective trial. J Craniomaxillofac Surg. 1999;27(1):30-7. doi:10.1016/s1010-5182(99)80007-9. | Observational-Dx and Tx | 45 | A prospective trial with the objective of assessing the utility of biomodelling in complex surgery has been performed. | An assessment protocol was used to test the hypothesis that 'biomodels in addition to standard imaging had greater utility in the surgery performed than the standard imaging alone'. Biomodels significantly improved operative planning (images 44.09%, images with biomodel 82.21%, P < .01) and diagnosis (images 65.63%, images with biomodel 95.23%, P < .01). Biomodels were found to improve measurement accuracy significantly (image measurement error 44.14%, biomodel measurement error 7.91%, P < .05). Surgeons estimated that the use of biomodels reduced operating time by a mean of 17.63% and were cost effective at a mean price of $1031 AUS. Patients found the biomodels to be helpful for informed consent (images 63.53%, biomodels 88.54%, P < .001). | 2 |
| 9 | Okonogi S, Kondo K, Harada N, Masuda H, Nemoto M, Sugo N. Operative simulation of anterior clinoidectomy using a rapid prototyping model molded by a three-dimensional printer. Acta Neurochir (Wien). 2017;159(9):1619-26. doi:10.1007/s00701-017-3202-4. | Observational-Dx and Tx | 51 | The objective of this study was to evaluate anatomical reproduction of the 3D synthetic image and intraosseous region after AC in the RP model. In addition, the usefulness of the RP model for operative simulation was investigated. | The RP model reproduced the region in the vicinity of the ACP in the 3D synthetic image, including the intraosseous region, at a high accuracy. In addition, drilling of the RP model was a useful operative simulation method of AC. | 3 |
| 10 | Kondo K, Harada N, Masuda H, et al. A neurosurgical simulation of skull base tumors using a 3D printed rapid prototyping model containing mesh structures. *Acta Neurochir (Wien).* 2016;158(6):1213-1219. | Review/Other-Dx | Not clearly stated | The objectives of this study were to develop an RP model in which a skull base tumor was simulated using mesh, and to investigate its usefulness for surgical simulations by evaluating the visibility of its deep regions. | The internal carotid artery, basilar artery, and brain stem and the positional relationships of these structures with the tumor were significantly more visible in the RP models with mesh tumors than in the RP models with solid or no tumors. | 4 |
| 11 | Abe M, Tabuchi K, Goto M, Uchino A. Model-based surgical planning and simulation of cranial base surgery. Neurol Med Chir (Tokyo). 1998;38(11):746-50; discussion 50-1. doi:10.2176/nmc.38.746. | Review/Other-Tx | 7 | Skull models were utilized for neurosurgical planning and simulation in the seven patients with cranial base lesions that were difficult to remove. | In preoperative simulations, hand-made models of the tumors, major vessels and nerves were placed in the skull models. Step-by-step simulation of surgical procedures was performed using actual surgical tools. The advantages of using skull models to plan and simulate cranial base surgery include a better understanding of anatomic relationships, preoperative evaluation of the proposed procedure, increased understanding by the patient and family, and improved educational experiences for residents and other medical staff. The disadvantages of using skull models include the time and cost of making the models. | 4 |
| 12 | Lin QS, Lin YX, Wu XY, Yao PS, Chen P, Kang DZ. Utility of 3-Dimensional-Printed Models in Enhancing the Learning Curve of Surgery of Tuberculum Sellae Meningioma. *World Neurosurg.* 2018;113:e222-e231. | Review/Other-Dx | 4 | To investigate the value of 3-dimensional (3D)-printed models with pathologic entities in enhancing the learning curve of surgery of tuberculum sellae meningioma. | A total of 42 new trainees were recruited, of whom 22 were in the 3D group and 20 in the atlas group. The baseline data were not significantly different. The difference of pre-test score was not significant, either. However, the post-test score was significantly greater in the 3D group (P = 0.005), and the change in score was also significantly greater in the 3D group (P < 0.001). In accordance with the objective test, the subjective survey through a questionnaire from participants in the 3D group showed that 3D models significantly promoted the learning curve of this kind of complex skull base surgery. | 4 |
| 13 | Hsieh TY, Cervenka B, Dedhia R, Strong EB, Steele T. Assessment of a Patient-Specific, 3-Dimensionally Printed Endoscopic Sinus and Skull Base Surgical Model. *JAMA Otolaryngol Head Neck Surg.* 2018;144(7):574-579. | Review/Other-Dx | 0 | To describe the development of 3D-printed sinus and skull base models for use in endoscopic skull base surgery. | Seven otolaryngology residents (3 postgraduate year [PGY]-5 residents, 2 PGY-4 residents, 1 PGY-3 resident, and 1 PGY-2 resident) and 2 attending physicians evaluated the haptic feedback of the 3D model. Computed tomographic comparison demonstrated a less than 5% difference between patient and 3D model measurements. Image-guided navigation confirmed accuracy of 13 landmarks to within 1 mm. Likert scores were a mean (SD) of 4.00 (0.71) for overall procedural anatomical accuracy and 4.67 (0.5) for haptic feedback. | 4 |
| 14 | Shah KJ, Peterson JC, Beahm DD, Camarata PJ, Chamoun RB. Three-Dimensional Printed Model Used to Teach Skull Base Anatomy Through a Transsphenoidal Approach for Neurosurgery Residents. *Oper Neurosurg (Hagerstown).* 2016;12(4):326-329. | Review/Other-Dx | Not clearly stated | To demonstrate that stereolithography, or 3-dimensional (3-D) printing, is a useful educational tool for neurosurgery residents to learn skull base anatomy. | A maximum score of 8 points was possible if all structures were identified correctly. Group A had mean scores of 2.75 on initial testing compared with 5 after the lecture (P = .041 using 2-tailed t test). Group B had mean scores of 2.75 on initial testing compared with 7.5 after the lecture and 3-D model simulation (P = .002). When comparing mean scores after formal teaching in groups A and B, 5 vs 7.5 were obtained for lecture only vs lecture and 3-D model simulation, respectively (P = .031). | 4 |
| 15 | Sanchez-Gomez S, Herrero-Salado TF, Maza-Solano JM, Ropero-Romero F, Gonzalez-Garcia J, Ambrosiani-Fernandez J. Improved planning of endoscopic sinonasal surgery from 3-dimensional images with Osirix(R) and stereolithography. Acta Otorrinolaringol Esp. 2015;66(6):317-25. doi:10.1016/j.otorri.2014.10.002. | Observational-Tx | 7 | The aim of the study was to validate the utility of Osirix® and stereolithography in improving endoscopic sinonasal surgery planning. | Using Osirix® and stereolithography, a greater number of anatomical structures were identified and this was done faster, with a statistically-significant clinical-radiological correla- tion (*P*<.01) compared with 2D CT plates. With a share of more than 75% of surgery performed by residents, surgical time was reduced by 38 ± 12.3 min in CRS and 42 ± 27.9 in sinonasal polypo- sis. The fourth-year residents reached 100% surgical competence in critical surgical milestones with 16 surgeries (CI 12-19). | 3 |
| 16 | [Shinomiya A, Shindo A, Kawanishi M, Miyake K, Nakamura T, Matsubara S et al. Usefulness of the 3D virtual visualization surgical planning simulation and 3D model for endoscopic endonasal transsphenoidal surgery of pituitary adenoma: Technical report and review of literature. Interdisciplinary Neurosurgery. 2018;13:13-9. doi:https://doi.org/10.1016/j.inat.2018.02.002.](doi:https://doi.org/10.1016/j.inat.2018.02.002.) | Review/Other-Tx | 12 | The purpose of this research was to investigate the usefulness of three-dimensional print models (3D models) in endoscopic endonasal transsphenoidal surgery (EeTSS) for pituitary adenoma. | We manufactured a 3D virtual surgical planning image using the patient's Digital Imaging and Communication in Medicine dataset. Based on the image, a life-size model was created by a 3D printer using multi-material acrylic-based resin. Our results suggest that such 3D models could be useful in EeTSS for pituitary adenoma. | 4 |
| 17 | Lin J, Zhou Z, Guan J, Zhu Y, Liu Y, Yang Z et al. Using Three-Dimensional Printing to Create Individualized Cranial Nerve Models for Skull Base Tumor Surgery. World Neurosurg. 2018;120:e142-e52. doi:10.1016/j.wneu.2018.07.236. | Review/Other-Tx | 2 | Using three-dimensional (3D) printing to create individualized patient models of the skull base, the optic chiasm and facial nerve can be previsualized to help identify and protect these structures during tumor removal surgery. | The 3D printed personalized skull base tumor solid models contained information regarding the skull, brain tissue, blood vessels, cranial nerves, tumors, and other associated structures. The sphenoid sinus anatomy, saddle area, and cerebellopontine angle region could be visually displayed, and the spatial relationship between the tumor and the cranial nerves and important blood vessels was clearly defined. The models allowed for simulation of the operation, prediction of operative details, and verification of accuracy of cranial nerve reconstruction during the operation. Questionnaire assessment showed that neurosurgeons highly valued the accuracy and usefulness of these skull base tumor models. | 4 |
| 18 | Huang X, Liu Z, Wang X, Li XD, Cheng K, Zhou Y et al. A small 3D-printing model of macroadenomas for endoscopic endonasal surgery. Pituitary. 2019;22(1):46-53. doi:10.1007/s11102-018-0927-x. | Observational-Tx | 20 | This paper examines the application of 3D printing technology in the endoscopic endonasal approach for the treatment of macroadenomas. | The 10 patients who received the service had a successful 3D printed model of their tumors, it shows the anatomy of sphenoid sinus, tumor location which were in good agreement with our intraoperative observations. The 10 patients who received the service had a less operation time (127.0 ± 15.53 vs. 143.40 ± 17.89), blood loss (159.90 ± 12.31 vs. 170.00 ± 29.06) and less postoperative complication rate (20% vs. 40%). the design time of the 3D images varies 2 h 10 min to 4 h 32 min. the printing time of the 3D models varies 10 h 12 min to 22 h 34 min. | 3 |
| 19 | Zheng JP, Li CZ, Chen GQ. Multimaterial and multicolor 3D-printed model in training of transnasal endoscopic surgery for pituitary adenoma. *Neurosurg Focus.* 2019;47(6):E21. | Review/Other-Dx | 3 | The aim of the present study was to investigate the practical value of a multimaterial and multicolor 3D-printed model in anatomical teaching, surgical training, and preoperative planning of transnasal endoscopic surgery for pituitary adenoma. | The multimaterial and multicolor model was superior to the monomaterial models in surgical training for exposing the vidian nerve (Fisher test; p < 0.05). In addition, the multimaterial and multicolor model was superior to the monomaterial models in anatomical teaching and preoperative planning (Friedman test; p < 0.05). | 4 |
| 20 | Waran V, Menon R, Pancharatnam D, et al. The creation and verification of cranial models using three-dimensional rapid prototyping technology in field of transnasal sphenoid endoscopy. *Am J Rhinol Allergy.* 2012;26(5):132-136. | Review/Other-Dx | Not clearly stated | A technique using an industrial rapid prototyping process by three-dimensional (3D) printing was developed, from which accurate spatial models of the nasal cavity, paranasal sinuses (sphenoid sinus in particular), and intrasellar/pituitary pathology were produced, according to the parameters of an individual patient. | It was possible to register, validate, and navigate accurately on these models using commonly available navigation stations, matching accurately the anatomy of the model to the IGS images. | 4 |
| 21 | Shen Z, Xie Y, Shang X, et al. The manufacturing procedure of 3D printed models for endoscopic endonasal transsphenoidal pituitary surgery. *Technol Health Care.* 2020;28(S1):131-150. | Review/Other-Dx | Not clearly stated | CT images are used as the source data of 3D printing. The data obtained directly from the CT machine has limited accuracy, which cannot be printed without processing. Some commercial platforms can help build an accurate model but the cost and customization are not satisfactory. In this situation, a tactile, precise and low-cost 3D model is highly desirable. | This study proposes a practical and cost-effective method to obtain the corrected digital model and produce the 3D printed skull with complete structures of nasal cavity, sellar region and different levels of pituitary tumors. The model is used for the endoscopic endonasal transsphenoidal pituitary surgery preparation. | 4 |
| 22 | Guo F, Wang G, Suresh V, Xu D, Zhang X, Feng M et al. Clinical study on microsurgical treatment for craniopharyngioma in a single consecutive institutional series of 335 patients. Clin Neurol Neurosurg. 2018;167:162-72. doi:10.1016/j.clineuro.2018.02.034. | Observational-Dx and Tx | 45 | The aim of this study is to explore microsurgical outcomes of craniopharyngioma in 335 cases. | Gross total resection (GTR) was achieved in 265 cases (79.1%), subtotal resection (STR) was obtained in 70 cases (20.9%). The GTR rate was 81.93% in pediatric group and 78.17% in adult group respectively, no significant difference regarding the GTR rate was found in adult group compared with in pediatric group (p > 0.05). However, there was a noticeable difference in the elevated hypothalamic obesity in children group compared with in adult group after operation (p < 0.05). Multivariate analysis indicated that the tumor re- currence and surgical times played a negative role in the resection extent, the odds ratio and 95% confidence interval of the tumor recurrence and surgical times is [0.306 (0.155–0.603), (p < 0.01)] and [2.135 (1.101–4.142), (p < 0.05)] respectively. There was significant difference on panhypopituitarism between GTR and STR group (p < 0.05). However, No significant difference regarding the postoperative visual dysfunction and indepent quality of life respectively between GTR and STR group was found (p > 0.05). Additionally, there were no statistically significant differences for recurrence-free curves between GTR and STR plus adjuvant radiotherapy (p > 0.05). | 2 |
| 23 | Fernandez-Miranda JC, Hwang P, Grant G. Endoscopic Endonasal Surgery for Resection of Giant Craniopharyngioma in a Toddler-Multimodal Presurgical Planning, Surgical Technique, and Management of Complications: 2-Dimensional Operative Video. *Oper Neurosurg (Hagerstown).* 2020;19(1):E68-E69. | Review/Other- Tx | 1 | Virtual reality simulation and 3-dimensional printing were employed to evaluate whether the absence of pneumatization of the sinuses and the overall size of the nasal cavity could preclude effective surgical access. | The patient has made an extraordinary recovery with no neurological sequalae. | 4 |
| 24 | Oishi M, Fukuda M, Yajima N, Yoshida K, Takahashi M, Hiraishi T et al. Interactive presurgical simulation applying advanced 3D imaging and modeling techniques for skull base and deep tumors. J Neurosurg. 2013;119(1):94-105. doi:10.3171/2013.3.JNS121109. | Review/Other-Tx | 25 | the authors' goal was to report their novel presurgical simulation method applying interactive virtual simulation (IVS) using 3D computer graphics (CG) data and microscopic observation of color-printed plaster models based on these CG data in surgery for skull base and deep tumors. | In all patients, IVS provided detailed and realistic surgical perspectives of sufficient quality, thereby allowing surgeons to determine an appropriate and feasible surgical approach. Surgeons agreed that in 44% of the 25 operations IVS showed high utility (as indicated by a rating of "prominent") in comprehending 3D microsurgical anatomies for which reconstruction using only 2D images was complicated. Microscopic observation of color-printed plaster models in 12 patients provided further utility in confirming realistic surgical anatomies. | 4 |
| 25 | Westendorff C, Kaminsky J, Ernemann U, Reinert S, Hoffmann J. Image-guided sphenoid wing meningioma resection and simultaneous computer-assisted cranio-orbital reconstruction: technical case report. Neurosurgery. 2007;60(2 Suppl 1):ONSE173-4; discussion ONSE4. doi:10.1227/01.NEU.0000249235.97612.52. | Review/Other-Tx | 1 | Resection of large intraosseous sphenoid wing meningiomas is traditionally associated with significant morbidity. Rapid prototyping techniques have become widely used for treatment planning. Yet, the transfer of a treatment plan into the intraoperative situs strongly depends on the experience of the individual surgeon. | In the presented case report, the combination of computer-assisted planning using rapid prototyping techniques and image-guided surgery allowed for an extensive tumor resection precisely according to a preoperative treatment plan in a patient presenting with a large intraosseous sphenoid wing meningioma. | 4 |
| 26 | Luo J, Morrison DA, Hayes AJ, Bala A, Watts G. Single-Piece Titanium Plate Cranioplasty Reconstruction of Complex Defects. *J Craniofac Surg.* 2018;29(4):839-842. | Review/Other- Tx | 1 | In describing the first reported patient of combined skull and orbital roof reconstruction with a single-piece titanium plate cranioplasty, the authors present a new method of combined complex cranio-orbital reconstruction. | The patient underwent hemicraniectomy and tumor resection guided by a custom-made 3D-printed cutting guide. The surgical defect was reconstructed with the prefabricated titanium plate achieving a good functional and cosmetic result. | 4 |
| 27 | Muller A, Krishnan KG, Uhl E, Mast G. The application of rapid prototyping techniques in cranial reconstruction and preoperative planning in neurosurgery. J Craniofac Surg. 2003;14(6):899-914. doi:10.1097/00001665-200311000-00014. | Review/Other-Tx | 52 | The value of rapid prototype models of the skull in our craniofacial and neurosurgical practice was analyzed. | The utility of the stereolithographic models was assessed using the Gillespie scoring system. The esthetic and clinical outcomes were assessed by means of the esthetic outcome score and the Glasgow Outcome Score, respectively. Simulation of osteotomies for advancement plasty and craniofacial reassembly in the model before surgery in group I reduced operating time and intraoperative errors. In group II, the usefulness of the models depended directly on the size and configuration of the cranial defect. The planning of approaches to uncommon and complex skull base tumors (group III) was significantly influenced by the stereolithographic models. The esthetic outcome was pleasing. | 4 |
| 28 | Bullock P, Dunaway D, McGurk L, Richards R. Integration of image guidance and rapid prototyping technology in craniofacial surgery. Int J Oral Maxillofac Surg. 2013;42(8):970-3. doi:10.1016/j.ijom.2013.04.019. | Review/Other-Tx | 1 | This technical note demonstrates the benefits of preoperative planning, involving the use of rapid prototype models and rehearsal of the surgical procedure, using image-guided navigational surgery. | Optimum reconstruction of large defects can be achieved with this technique. | 4 |
| 30 | Kondo K, Nemoto M, Harada N, et al. Three-Dimensional Printed Model for Surgical Simulation of Combined Transpetrosal Approach. *World Neurosurg.* 2019;127:e609-e616. | Review/Other-Dx | 1 | We made a 3-dimensional (3D) printed petrous bone with color-coded anatomic sites and evaluated its usefulness as a model to practice drilling associated with combined transpetrosal surgery. | The anatomic reproducibility of both the 3D image and model was high, and the 3D model was considered good for drilling practice (P < 0.05). The error in the estimated distance between anatomic sites in the 3D model was significantly smaller than that of the 3D image (P < 0.0001). | 4 |
| 31 | Muelleman TJ, Peterson J, Chowdhury NI, Gorup J, Camarata P, Lin J. Individualized Surgical Approach Planning for Petroclival Tumors Using a 3D Printer. *J Neurol Surg B Skull Base.* 2016;77(3):243-248. | Review/Other-Dx and Tx | 3 | To determine the utility of three-dimensional (3D) printed models in individualized petroclival tumor resection planning by measuring the fidelity of printed anatomical structures and comparing tumor exposure afforded by different approaches. | Surgeons found the 3D models of each patient's skull and tumor useful for preoperative planning. Limitations of individual surgical approaches not identified through preoperative imaging were apparent after 3D models were evaluated. Significant variability in exposure was noted between models for similar or identical approaches. A notable drawback is that our printing process did not replicate mastoid air cells. | 4 |
| 32 | Panesar SS, Magnetta M, Mukherjee D, et al. Patient-specific 3-dimensionally printed models for neurosurgical planning and education. *Neurosurg Focus.* 2019;47(6):E12. | Review/Other-Dx and Tx | 4 | The goal of this study was to assess the clinical value of patient-specific 3D printed models for neurosurgical planning and education. | Life-sized, 3D printed models depicting bony, vascular, and neural pathology relevant to each case were successfully manufactured. A variety of commercially available software and hardware were used to create and print each model from radiological sequences. The models for the adult cases were printed in separate pieces, which had to be painted by hand, and could be disassembled for detailed study, while the model for the pediatric case was printed as a single piece in separate-colored resins and could not be disassembled for study. Two of the models were used for patient education, and all were used for presurgical planning by the surgeon. | 4 |
| 33 | Kosterhon M, Neufurth M, Neulen A, et al. Multicolor 3D Printing of Complex Intracranial Tumors in Neurosurgery. *J Vis Exp.* 2020(155). | Review/Other- Tx | Not clearly stated | A step-by-step guide is provided, demonstrating the fusion of different cross-sectional imaging data sets, segmentation of anatomical structures, and creation of a virtual model. | This method allows highly accurate reproduction of patient-specific anatomy as shown in a series of 3D-printed petrous apex chondrosarcomas. | 4 |
| 34 | Pijpker PAJ, Wagemakers M, Kraeima J, Vergeer RA, Kuijlen JMA, Groen RJM. Three-Dimensional Printed Polymethylmethacrylate Casting Molds for Posterior Fossa Reconstruction in the Surgical Treatment of Chiari I Malformation: Technical Note and Illustrative Cases. *World Neurosurg.* 2019;129:148-156. | Review/Other- Tx | 3 | To describe a new method for cranial reconstruction after posterior fossa craniectomy in the surgical treatment of Chiari 1 malformation through a technical note and presentation of 3 illustrative cases. | The accuracy of implant fabrication was found to be excellent, particularly when PMMA is introduced into the mold in a malleable state. In all 3 clinical cases, the implants were fabricated and positioned with success. Postoperative analysis revealed that accurate placement was achieved, with only minor deviation from the preoperative plan. | 4 |
| 35 | Liu JY, Man QW, Ma YQ, Liu B. I(125) brachytherapy guided by individual three-dimensional printed plates for recurrent ameloblastoma of the skull base. *Br J Oral Maxillofac Surg.* 2017;55(7):e38-e40. | Review/Other-Dx and Tx | 1 | We report a case of recurrent ameloblastoma of the base of the skull that was treated with I125 brachytherapy. | The outcome has been encouraging, with total disappearance of the tumour on positron emission tomography 18 months later. | 4 |
| 36 | Fernandes N, van den Heever J, Hoogendijk C, Botha S, Booysen G, Els J. Reconstruction of an Extensive Midfacial Defect Using Additive Manufacturing Techniques. *J Prosthodont.* 2016;25(7):589-594. | Review/Other- Tx | 1 | We present a clinical case involving a 33-year-old female patient presenting with a slow-growing, exophytic mass of the anterior maxilla. | An anatomical model of the hard tissues was manufactured via 3D printing. This model was used to design and manufacture a titanium frame (customized implant) for the patient. The frame was then fixated and secured intraoperatively with 21 cortical screws. A maxillary denture and silicone facial prosthesis were also made to fit onto this frame. This is the first known case where additive manufacturing, via the use of rapid prototyping and 3D printing, was employed to manufacture a facial prosthesis. | 4 |
| 37 | Broeckx CE, Maal TJJ, Vreeken RD, Bos RRM, Ter Laan M. Single-Step Resection of an Intraosseous Meningioma and Cranial Reconstruction: Technical Note. *World Neurosurg.* 2017;108:225-229. | Review/Other- Tx | 1 | In this technical note, we describe a new technique for this procedure to ensure a precise resection and optimal fit of the implant in a patient with an intraosseous meningioma. | The planned resection was achieved, and the implant could be fitted without need for further adjustments to the resection border. | 4 |
| 38 | Guo XY, He ZQ, Duan H, et al. The utility of 3-dimensional-printed models for skull base meningioma surgery. *Ann Transl Med.* 2020;8(6):370. | Review/Other- Tx | 35 | A retrospective study of 35 patients (3D group: 19 patients and non-3D group: 16 patients) with skull base meningioma was conducted. | The 3D-printed model can visually display the relationship of different structures, including the skull, blood vessels, cranial nerves, and tumors. The surgeon should select the proper surgical approaches before surgery through the model and pay attention to protecting the important structures during the operation. According to the models, the surgeon should cut off the blood supply to the tumor to reduce intraoperative bleeding. For patients with skull base bone destruction, the skull base repair should be prepared in advance. Patients and their families should have a thorough understanding of the disease through the model, and there should be effective communication between doctors and patients. | 4 |
| 39 | Rashim K, Verma Pawan K, Sinha VD. Increasing the safety of surgical treatment for complex Cranio-vertebral anomalies using customized 3D printed models. *J Clin Neurosci.* 2018;48:203-208. | Review/Other- Tx | 13 | The customised 3D printed model of CV junction region of the patient can be used for studying the anatomy and relationship of vertebral artery to the C1-C2 joint before the actual surgery. | Post surgery, twelve out of thirteen patients showed significant clinical and radiological improvement. We did not had any misplaced screws or vertebral artery injury. | 4 |
| 40 | Yuan T, Jia G, Yang L, Xu D, Zhang J, Liu Q. Occipitocervical fusion combined with 3-dimensional navigation and 3-dimensional printing technology for the treatment of atlantoaxial dislocation with basilar invagination: A case report. *Medicine (Baltimore).* 2020;99(5):e18983. | Review/Other- Tx | 1 | We present a case of BI treated with posterior-only occipitocervical fusion combined with 3D printing technology and 3D navigation system to reduce the risk of surgical complications. | The patient's walking disorder was resolved and he was able to walk approximately 100 m by himself when he was allowed to get up and move around with the help of a neck brace. At 6 months postoperatively, the patient reported that the numbness of the limbs was reduced, and he could walk >500 m by himself. | 4 |
| 41 | Goel A, Jankharia B, Shah A, Sathe P. Three-dimensional models: an emerging investigational revolution for craniovertebral junction surgery. *J Neurosurg Spine.* 2016;25(6):740-744. | Review/Other- Tx | 11 | The authors present their experience with the emerging technology of 3D model acquisition for surgery in 11 cases of complex craniovertebral junction region anomalies. | The sizes of the plates and screws to be used and the angle of insertion of the screws were calculated based on the data from the models. The model was scaled to actual size and was kept beside the operating surgeon in its anatomical position during surgery. | 4 |
| 42 | Wang J, Zhu C, Xia H. Management of Unique Basilar Invagination Combined with C1 Prolapsing into the Foramen Magnum in Children: Report of 2 Cases. *World Neurosurg.* 2019;127:92-96. | Review/Other- Tx | 2 | We present 2 rare cases of BI combined with C1 prolapsing into the FM. | We adopted different surgical strategies with satisfying outcome for these patients. We deem that the treatment of unique BI should be individualized according to the different image characteristics. The image-based modern rapid prototyping and 3D printed techniques can provide invaluable information in presurgical planning for complex craniovertebral junction anomalies. | 4 |
| 43 | Narayanan V, Narayanan P, Rajagopalan R, et al. Endoscopic skull base training using 3D printed models with pre-existing pathology. *Eur Arch Otorhinolaryngol.* 2015;272(3):753-757. | Review/Other- Tx | 5 | This paper aims to assess the ease of learning endoscopic skull base exposure and drilling techniques using an anatomically accurate physical model with a pre-existing pathology (i.e., basilar invagination) created from actual patient data. | The participants found the models suitable for learning registration, navigation and skull base drilling techniques. All participants also found the deep structures to be accurately represented spatially as confirmed by the navigation system. | 4 |
| 44 | Du YQ, Qiao GY, Yin YH, Li T, Tong HY, Yu XG. Usefulness of 3D Printed Models in the Management of Complex Craniovertebral Junction Anomalies: Choice of Treatment Strategy, Design of Screw Trajectory, and Protection of Vertebral Artery. *World Neurosurg.* 2020;133:e722-e729. | Review/Other- Tx | 21 | To evaluate the usefulness of 3-dimensional (3D) printed models as an aid for the treatment of complex CVJ anomalies. | Direct posterior reduction and atlantoaxial fixation were achieved in 19 patients. Transoral odontoidectomy followed by posterior fixation was implemented for 2 patients with vertical facet joint and rotational dislocation. All screws were safely inserted with no complication, and 90% patients achieved a >60% reduction of both horizontal and vertical dislocation. Clinical symptoms improved in all patients, with the averaged Japanese Orthopedic Association scores increasing from 11.14 to 14.43 (P < 0.01). | 4 |
| 45 | He S, Ye C, Zhong N, Yang M, Yang X, Xiao J. Customized anterior craniocervical reconstruction via a modified high-cervical retropharyngeal approach following resection of a spinal tumor involving C1-2/C1-3. *J Neurosurg Spine.* 2019:1-9. | Observational-Tx | 7 | The authors attempted to introduce a novel, customized, anterior craniocervical reconstruction between the occipital condyles and inferior vertebrae through a modified high-cervical retropharyngeal approach (mHCRA) in addressing C1-2/C1-3 spinal tumors. | The mean age of the 7 patients in the study was 47.6 ± 19.0 years (range 12-72 years) when referred to the authors' center. Six patients (85.7%) had recurrent tumor status, and the interval from primary to recurrence status was 53.0 ± 33.7 months (range 24-105 months). Four patients (57.1%) were diagnosed with a spinal tumor involving C1-3, and 3 patients (42.9%) with a C1-2 tumor. For the anterior procedure, the mean surgical duration and average blood loss were 4.1 ± 0.9 hours (range 3.0-6.0 hours) and 558.3 ± 400.5 ml (range 100-1300 ml), respectively. No severe perioperative complications occurred, except 1 patient with transient dysphagia. The mean pre- and postoperative visual analog scale scores were 8.0 ± 0.8 (range 7-9) and 2.4 ± 0.5 (range 2.0-3.0; p < 0.001), respectively, and the mean improvement rate of cervical spinal cord function was 54.7% ± 13.8% (range 42.9%-83.3%) based on the modified Japanese Orthopaedic Association scale score (p < 0.001). Circumferential instrumentation was in good position and no evidence of disease was found at the mean follow-up of 14.8 months (range 7.3-24.2 months). | 3 |
| 46 | Pacione D, Tanweer O, Berman P, Harter DH. The utility of a multimaterial 3D printed model for surgical planning of complex deformity of the skull base and craniovertebral junction. *J Neurosurg.* 2016;125(5):1194-1197. | Review/Other- Tx | 1 | Utilizing advanced 3D printing techniques, a multimaterial model was created for the surgical planning of a complex deformity of the skull base and craniovertebral junction. | This patient-specific model was invaluable in choosing the most effective approach and correction strategy, which was not readily apparent from standard 2D imaging. Advanced 3D multimaterial printing provides a cost-effective method of presurgical planning, which can also be used for both patient and resident education. | 4 |
| 47 | Barber SR, Wong K, Kanumuri V, et al. Augmented Reality, Surgical Navigation, and 3D Printing for Transcanal Endoscopic Approach to the Petrous Apex. *OTO Open.* 2018;2(4):2473974X18804492. | Review/Other- Tx | Not clearly stated | We aim to (1) use a 3D-printed patient-specific physical model with lateral skull base navigation for preoperative planning, (2) review anatomy virtually via augmented reality (AR), and (3) compare physical and virtual models to intraoperative findings in a challenging case of a symptomatic petrous apex cyst. | Virtual and physical models adequately addressed details of endoscopic surgery, including avoidance of critical structures. | 4 |
| 48 | Chae R, Sharon JD, Kournoutas I, et al. Replicating Skull Base Anatomy With 3D Technologies: A Comparative Study Using 3D-scanned and 3D-printed Models of the Temporal Bone. *Otol Neurotol.* 2020;41(3):e392-e403. | Observational-Dx | 0 | 3D technologies, including structured light scanning (SLS), microcomputed tomography (micro-CT), and 3D printing, are valuable tools for reconstructing temporal bone (TB) models with high anatomical fidelity and cost-efficiency. | Significant differences between the physical skulls and virtual models were observed for 11 of 14 parameters (p < 0.0036), with the greatest mean difference in the length of petrous ridge (2.85 mm) and smallest difference in the diameter of stylomastoid foramen (0.67 mm). In the secondary analysis, greater mean differences were observed between TBi and virtual models than between TBi and 3D-printed models. | 3 |
| 49 | Ritacco LE, Di Lella F, Mancino A, Gonzalez Bernaldo de Quiros F, Boccio C, Milano FE. 3D Printed Models and Navigation for Skull Base Surgery: Case Report and Virtual Validation. *Stud Health Technol Inform.* 2015;216:1025. | Review/Other-Dx | 1 | The aim of this project is to propose a comparative validation method to enable physicians to evaluate differences between a virtual planned approach trajectory and a real executed course. | This project is focused on decoding data in order to obtain numerical values so as to establish the quality of surgical procedures, specifically in a patient with cholesteatoma. | 4 |
| 50 | Rose AS, Kimbell JS, Webster CE, Harrysson OL, Formeister EJ, Buchman CA. Multi-material 3D Models for Temporal Bone Surgical Simulation. *Ann Otol Rhinol Laryngol.* 2015;124(7):528-536. | Review/Other- Tx | Not clearly stated | A simulated, multicolor, multi-material temporal bone model can be created using 3-dimensional (3D) printing that will prove both safe and beneficial in training for actual temporal bone surgical cases. | The models produced for this study demonstrate significant anatomic detail and a likeness to human cadaver specimens for drilling and dissection. | 4 |
| 51 | McMillan A, Kocharyan A, Dekker SE, et al. Comparison of Materials Used for 3D-Printing Temporal Bone Models to Simulate Surgical Dissection. *Ann Otol Rhinol Laryngol.* 2020:3489420918273. | Review/Other- Tx | Not clearly stated | To identify 3D-printed temporal bone (TB) models that most accurately recreate cortical mastoidectomy for use as a training tool by comparison of different materials and fabrication methods. | Surgical drilling demonstrated that FLW models created by FDM as well as PC and Photo models generated using photopolymerization more closely recreated cortical mastoidectomy compared to ABS models. ABS generated odor and did not represent the anatomy accurately. Blue resin performed poorly in simulation, likely due to its dark color and translucent appearance. | 4 |
| 52 | Bone TM, Mowry SE. Content Validity of Temporal Bone Models Printed Via Inexpensive Methods and Materials. *Otol Neurotol.* 2016;37(8):1183-1188. | Observational-Dx | 0 | Computed tomographic (CT) scans of the 3-D printed temporal bone models will be within 15% accuracy of the CT scans of the cadaveric temporal bones. | Mean pixel difference between the cadaver and model scans was 14.25 ± 2.30% at the four selected CT slices. Mean cortical bone width difference and mean external auditory canal width difference were 0.58 ± 0.66 mm and 0.55 ± 0.46 mm, respectively. Expert raters felt the mastoid air cells were well represented (2.5 ± 0.5), while middle ear and otic capsule structures were not accurately rendered (all averaged <1.8). | 3 |
| 53 | Takahashi K, Morita Y, Ohshima S, et al. Creating an Optimal 3D Printed Model for Temporal Bone Dissection Training. *Ann Otol Rhinol Laryngol.* 2017;126(7):530-536. | Review/Other- Tx | Not clearly stated | The objective of this study was to overcome these problems and create a temporal bone model that would be useful both as a training tool and for preoperative simulation. | Macroscopic and endoscopic inspection, CT images, and assessment by surgeons were in agreement in terms of reproducibility of model structures. Most structures could be reproduced, but the stapes, tympanic sinus, and mastoid air cells were unsatisfactory. Perioperative tactile sensation of the model was excellent. | 4 |
| 54 | Rose AS, Webster CE, Harrysson OL, Formeister EJ, Rawal RB, Iseli CE. Pre-operative simulation of pediatric mastoid surgery with 3D-printed temporal bone models. *Int J Pediatr Otorhinolaryngol.* 2015;79(5):740-744. | Review/Other- Tx | 1 | We aim to 3-dimensionally print customized prostheses to resurface or occlude bony SCD defects. | The simulation allowed the surgical team to appreciate the child's unusual temporal bone anatomy as well as any challenges that might arise in the safety of the temporal bone laboratory, prior to actual surgery in the operating room (OR). There was minimal variability, in terms of absolute distance (mm) and relative distance (%), in measurements between anatomic landmarks obtained from the patient intra-operatively, the pre-operative CT scan and the 3D-printed models. | 4 |
| 55 | Mowry SE, Jammal H, Myer Ct, Solares CA, Weinberger P. A Novel Temporal Bone Simulation Model Using 3D Printing Techniques. *Otol Neurotol.* 2015;36(9):1562-1565. | Review/Other- Tx | Not clearly stated | An inexpensive temporal bone model for use in a temporal bone dissection laboratory setting can be made using a commercially available, consumer-grade 3D printer. | The created model was felt to be an accurate representation of a human temporal bone. All raters felt strongly this would be a good training model for junior residents or to simulate difficult surgical anatomy. Material cost for each model was $1.92. | 4 |
| 56 | Suzuki M, Hagiwara A, Ogawa Y, Ono H. Rapid-prototyped temporal bone and inner-ear models replicated by adjusting computed tomography thresholds. *J Laryngol Otol.* 2007;121(11):1025-1028. | Review/Other- Tx | Not clearly stated | This study aimed to investigate the validity of adjusting computed tomography thresholds in order to replicate a temporal bone model suitable for dissection training and education. | The model could be shaved, using surgical instruments, in the same manner as during real surgery. The stapes could be reproduced, making this model even more realistic than a previous version. The inner ear was recreated, along with the surrounding bony wall and the ossicles. | 4 |
| 57 | Haffner M, Quinn A, Hsieh TY, Strong EB, Steele T. Optimization of 3D Print Material for the Recreation of Patient-Specific Temporal Bone Models. *Ann Otol Rhinol Laryngol.* 2018;127(5):338-343. | Review/Other- Tx | 0 | Identify the 3D printed material that most accurately recreates the visual, tactile, and kinesthetic properties of human temporal bone | Polyethylene terephthalate (PETG) had the highest average survey response for haptic feedback (HF) and appearance, scoring 8.3 (SD = 1.7) and 7.6 (SD = 1.5), respectively. The remaining plastics scored as follows for HF and appearance: polylactic acid (PLA) averaged 7.4 and 7.6, acrylonitrile butadiene styrene (ABS) 7.1 and 7.2, polycarbonate (PC) 7.4 and 3.9, and nylon 5.6 and 6.7. | 4 |
| 58 | Freiser ME, Ghodadra A, Hart L, Griffith C, Jabbour N. Safety of Drilling 3-Dimensional-Printed Temporal Bones. *JAMA Otolaryngol Head Neck Surg.* 2018;144(9):797-801. | Observational-Dx | 0 | To determine the level of exposure to airborne contaminants when conducting high-speed drilling on 3-D-printed models and to explore whether there is a need for exposure control measures. | Results of the VOC sample were less than detection limits except for isopropyl alcohol at 0.24 ppm for PAR. The TP samples were less than the detection limit of 1.4 mg/m3. The results are below all applicable OSHA Action Levels and Permissible Exposure Limits for all contaminants sampled for. | 3 |
| 59 | Freiser ME, Ghodadra A, Hirsch BE, McCall AA. Evaluation of 3D Printed Temporal Bone Models in Preparation for Middle Cranial Fossa Surgery. *Otol Neurotol.* 2019;40(2):246-253. | Review/Other- Tx | 0 | Patient-specific 3D printed models are useful presurgical planning tools because they accurately represent the anatomy and drilling characteristics of the middle cranial fossa (MCF) approach to the internal auditory canal (IAC). | Drilling the model was favorably rated (median score 9.2; range 7.3-9.6) for its ability to provide surgeons with an accurate mental image of the corresponding cadaveric anatomy. Overall similarity of feel of drilling the model in comparison to human bone was moderate (median 7.6; range 6.6-9.0). Surgeons would use this model to prepare for future cases (median 9.4; range 5.1-9.9) and felt it had excellent utility for training purposes (median 9.3; range 8.4-9.9). | 4 |
| 60 | Nguyen Y, Mamelle E, De Seta D, Sterkers O, Bernardeschi D, Torres R. Modifications to a 3D-printed temporal bone model for augmented stapes fixation surgery teaching. *Eur Arch Otorhinolaryngol.* 2017;274(7):2733-2739. | Review/Other- Tx | 0 | The goal of this work was to adapt such an artificial temporal bone to aid the teaching of otosclerosis surgery and to evaluate this tool. | No statistically significant differences were observed between the junior and senior groups for time taken to perform the tasks and the forces applied to the incus during crimping and placement of the prosthesis. | 4 |
| 61 | Hochman JB, Rhodes C, Wong D, Kraut J, Pisa J, Unger B. Comparison of cadaveric and isomorphic three-dimensional printed models in temporal bone education. *Laryngoscope.* 2015;125(10):2353-2357. | Review/Other- Tx | Not clearly stated | The purpose of this study is to determine if resident training level can be distinguished on the basis of performance employing a printed temporal bone model, graded by a previous validated scale. | ANOVA revealed significant performance differences between the junior/intermediate and junior/senior PGY cohorts. No difference was observed between intermediate/senior cohorts on the basis of PGY or subjective temporal bone dissection experience. Clustering aspects of the scale with specific focus on thinning tasks found a similar outcome to the composite scale scores.Subjective experience judged printed bone to be similar to cadaveric in drill-bone interaction. Participants believed the simulation would improve surgical performance, comfort with actual patients, and operative speed. | 4 |
| 62 | Hochman JB, Sepehri N, Rampersad V, et al. Mixed reality temporal bone surgical dissector: mechanical design. *J Otolaryngol Head Neck Surg.* 2014;43:23. | Review/Other- Dx | 0 | This paper introduces a mixed reality model, where the effective elements of both simulations are combined; haptic rendering of soft tissue directly interacts with a printed bone model. | Testing illustrated the effectiveness of gravity cancellation. Additionally, the system exhibited excellent performance given random inputs and during the drill's passage between real and virtual components of the model. No issues with registration at model boundaries were encountered. | 4 |
| 63 | Da Cruz MJ, Francis HW. Face and content validation of a novel three-dimensional printed temporal bone for surgical skills development. *J Laryngol Otol.* 2015;129 Suppl 3:S23-29. | Review/Other- Tx | Not clearly stated | To assess the face and content validity of a novel synthetic, three-dimensional printed temporal bone for surgical skills development and training. | Trainees' experiences of the synthetic temporal bone were analysed in terms of four domains: anatomical realism, usefulness as a training tool, task-based usefulness and overall reactions. Responses across all domains indicated a high degree of acceptance, suggesting that the three-dimensional printed temporal bone was a useful tool in skills development. | 4 |
| 64 | Cohen J, Reyes SA. Creation of a 3D printed temporal bone model from clinical CT data. *Am J Otolaryngol.* 2015;36(5):619-624. | Review/Other- Dx | Not clearly stated | Generate and describe the process of creating a 3D printed, rapid prototype temporal bone model from clinical quality CT images. | Mastoid air cells had retained scaffolding material in the initial versions. This required modifying the model to allow drainage of the scaffolding material. External auditory canal dimensions were similar to those measured from the clinical data. Malleus, incus, oval window, round window, promontory, horizontal semicircular canal, and mastoid segment of the facial nerve canal were identified in all models. The stapes was only partially formed in two models and absent in the third. Qualitative feel of the ABS plastic was softer than bone. The pate produced by drilling was similar to bone dust when appropriate irrigation was used. | 4 |
| 65 | Skrzat J, Zdilla MJ, Brzegowy P, Holda M. 3 D printed replica of the human temporal bone intended for teaching gross anatomy. *Folia Med Cracov.* 2019;59(3):23-30. | Review/Other- Tx | Not clearly stated | This report details the production of a durable physical replica of the adult human temporal bone, manufactured using 3D printing technology. | Both the virtual and physical 3D models accurately reproduced the surface anatomy of the temporal bone. | 4 |
| 66 | Bento RF, Rocha BA, Freitas EL, Balsalobre FA. Otobone ((R)) : Three-dimensional printed Temporal Bone Biomodel for Simulation of Surgical Procedures. *Int Arch Otorhinolaryngol.* 2019;23(4):e451-e454. | Review/Other- Tx | 1 | Developing a technique to produce temporal bone models that allow them to maintain the external and internal anatomical features faithful to the natural bone. | After dissection, the lead author evaluated the plasticity of the part and its similarity in drilling a natural bone as grade "4" on a scale of 0 to 5, in which 5 is the closest to the natural bone and 0 the farthest from the natural bone. All structures proposed in the method were found with the proposed color. | 4 |
| 67 | Gadaleta DJ, Huang D, Rankin N, et al. 3D printed temporal bone as a tool for otologic surgery simulation. *Am J Otolaryngol.* 2020;41(3):102273. | Review/Other- Tx | 0 | In this face validity study, we discuss the fabrication and utility of an affordable, computed tomography (CT)-based, anatomy-accurate, 3-dimensional (3D) printed temporal bone models for junior otolaryngology resident training. | The final result was an anatomically accurate (XYZ accuracy = 12.5, 12.5, 5 μm) 3D model of a temporal bone that was deemed to be appropriate in tactile feedback using the surgical drill. The total cost of the material required to fabricate the model was approximately $1.50. Participants found the 3D models overall to be similar to cadaveric temporal bones, particularly in overall value and safety. | 4 |
| 68 | Mick PT, Arnoldner C, Mainprize JG, Symons SP, Chen JM. Face validity study of an artificial temporal bone for simulation surgery. *Otol Neurotol.* 2013;34(7):1305-1310. | Review/Other- Dx | 0 | Using the rapid prototype (RP) technology, a physical construct of a human temporal bone was developed based on cadaveric tissue to permit simulated surgical training. The objective of the study was to test the face validity of the model. | In using a Likert scale between 1 and 5, results for anatomic accuracy were favorable, with the best scores for overall morphology (4.63) and for lateral structures within the bone (4.5). The poorest scores were for the semicircular canals (3.75) and chorda tympani (3.25). Scores for haptic realism were good as well. The average score for the question "overall, how valuable is the model as a surgical simulator" was 4.1. The experts felt that junior residents (PGY 1-3) would benefit most from this surgical education model. | 4 |
| 69 | Chauvelot J, Laurent C, Le Coz G, et al. Morphological validation of a novel bi-material 3D-printed model of temporal bone for middle ear surgery education. *Ann Transl Med.* 2020;8(6):304. | Observational-Dx | 0 | An original method is reported to quantify the model's ability to reproduce the complex anatomy of this region. | The evaluation of the segmentation and mesh correction steps revealed that the distance between both geometries was globally less that one millimeter for each anatomical region and close to zero for regions such as temporal bone, semicircular canals or facial nerve. The evaluation of the printing technique revealed mismatches of 0.045±0.424 mm for soft and -0.093±0.240 mm for hard tissues between the initial prepared geometry and the actual printed model. | 3 |
| 70 | Suzuki M, Ogawa Y, Kawano A, Hagiwara A, Yamaguchi H, Ono H. Rapid prototyping of temporal bone for surgical training and medical education. *Acta Otolaryngol.* 2004;124(4):400-402. | Review/Other- Tx | Not clearly stated | The aim of this study was to investigate the validity of a prototype temporal bone model for surgical training and education. | The model was as hard as real bone and surface structures were accurately reproduced. The model could be shaved using a surgical drill, burr and suction irrigator in the same way as a real bone. The malleus and incus were reproduced. The semicircular canals and the oval and round window niches were identified. Cavity structures, such as the semicircular canal, vestibule, antrum and air cells, were filled with powder which had to be removed using a pick and suction irrigator during dissection. A magnified model was useful for educating medical students. | 4 |
| 71 | Longfield EA, Brickman TM, Jeyakumar A. 3D Printed Pediatric Temporal Bone: A Novel Training Model. *Otol Neurotol.* 2015;36(5):793-795. | Review/Other- Tx | Not clearly stated | Our objective is to develop a pediatric temporal bone model. | Three models were produced and were evaluated. The models utilized multiple colors (white for bone, yellow for the facial nerve) and were of high quality. Two models were drilled as a proof of concept and found to be an acceptable facsimile of the patient's anatomy, rendering all necessary surgical landmarks accurately. The only negative comments pertaining to the 3D printed temporal bone as a training model were the lack of variation in hardness between cortical and cancellous bone, noting a tactile variation from cadaveric temporal bones. | 4 |
| 72 | Wong V, Unger B, Pisa J, Gousseau M, Westerberg B, Hochman JB. Construct Validation of a Printed Bone Substitute in Otologic Education. *Otol Neurotol.* 2019;40(7):e698-e703. | Review/Other- Tx | 0 | The purpose of this study is to determine if resident training level can be distinguished on the basis of performance employing a printed temporal bone model, graded by a previous validated scale. | Participants believed the simulation would improve surgical performance, comfort with actual patients, and operative speed. | 4 |
| 73 | Wanibuchi M, Noshiro S, Sugino T, et al. Training for Skull Base Surgery with a Colored Temporal Bone Model Created by Three-Dimensional Printing Technology. *World Neurosurg.* 2016;91:66-72. | Review/Other-Dx | Not clearly stated | A 3-dimensional temporal bone model for skull base surgical training was reconstructed via the use of a selective laser sintering technique, which is one of the 3-dimensional printing technologies. | The powder material was minimal, and the decisive structures were identified in color. | 4 |
| 74 | Hochman JB, Kraut J, Kazmerik K, Unger BJ. Generation of a 3D printed temporal bone model with internal fidelity and validation of the mechanical construct. *Otolaryngol Head Neck Surg.* 2014;150(3):448-454. | Review/Other- Tx | Not clearly stated | To generate a rapid-prototyped temporal bone model from computed tomography (CT) data with a specific focus on internal anatomic fidelity. | The printed bone models are highly realistic. Void space representation was excellent with 88% concordance between cadaveric bone and the resultant rapid-prototyped temporal bone model. Ultimately, cyanoacrylate with hydroquinone was determined to be the most appropriate infiltrant for both cortical and trabecular simulation. The mechanical properties of all tested infiltrants were similar to real bone. | 4 |
| 75 | Wu CT, Lee ST, Chen JF, Lin KL, Yen SH. Computer-aided design for three-dimensional titanium mesh used for repairing skull base bone defect in pediatric neurofibromatosis type 1. A novel approach combining biomodeling and neuronavigation. *Pediatr Neurosurg.* 2008;44(2):133-139. | Review/Other-Dx and Tx | 1 | In this report, we describe a novel surgical technique, combining computer-aided design, stereolithography and neuronavigation to repair a temporal base skull defect in a 16-year-old female patient with neurofibromatosis type 1. | The final graft of titanium mesh, which was intraoperatively fabricated based on the biomodel, was precisely orientated and securely fixed to the surrounding bone under frameless navigation. Long-term follow-up result proved this repair to be effective and durable. | 4 |
| 76 | Ahmed S, VanKoevering KK, Kline S, Green GE, Arts HA. Middle cranial fossa approach to repair tegmen defects assisted by three-dimensionally printed temporal bone models. *Laryngoscope.* 2017;127(10):2347-2351. | Review/Other- Tx | 5 | To explore the perioperative utility of three-dimensionally (3D)-printed temporal bone models of patients undergoing repair of lateral skull base defects and spontaneous cerebrospinal fluid leaks with the middle cranial fossa approach. | Five patients underwent the middle cranial fossa approach assisted by 3D-printed temporal bone models to repair tegmen defects and spontaneous cerebrospinal fluid leaks. No complications were encountered. The prefabricated dural repair grafts were easily placed and fit precisely onto the middle fossa floor without any additional modifications. All defects were covered as predicted by the 3D temporal bone models. At their postoperative visits, all five patients maintained resolution of their spontaneous cerebrospinal fluid leaks. | 4 |
| 77 | VanKoevering KK, Gao RW, Ahmed S, Green GE, Arts HA. A 3D-Printed Lateral Skull Base Implant for Repair of Tegmen Defects: A Case Series. *Otol Neurotol.* 2020. | Review/Other- Tx | 3 | To determine the feasibility of a patient-specific, three-dimensionally (3D)-printed reconstruction plate for repair of lateral skull base defects. | Real-time, intraoperative placement of the tegmen plate in our patients under 1 minute compared with nearly 60 minutes for standard surgical repair. Tegmen plates covered the defects and locked into place from contour matching without impinging on critical structures. Fit testing revealed flush-fitting plates to the cadaveric temporal bone surface with all gaps less than 500 μm. | 4 |
| 78 | Tai BL, Rooney D, Stephenson F, et al. Development of a 3D-printed external ventricular drain placement simulator: technical note. *J Neurosurg.* 2015;123(4):1070-1076. | Review/Other- Tx | 1 | In this paper, the authors present a physical model developed to simulate accurate external ventricular drain (EVD) placement with realistic haptic and visual feedbacks to serve as a platform for complete procedural training. | The resultant simulator provides realistic haptic feedback during a procedure, with visualization of catheter trajectory and fluid drainage. | 4 |
| 79 | Essayed WI, Unadkat P, Hosny A, et al. 3D printing and intraoperative neuronavigation tailoring for skull base reconstruction after extended endoscopic endonasal surgery: proof of concept. *J Neurosurg.* 2018;130(1):248-255. | Review/Other-Dx | 0 | In this study, the authors assessed the potential use of modern multimaterial 3D printing and neuronavigation to help model these extended defects and develop specifically tailored prostheses for reconstructive purposes. | Prostheses were created based on preoperative and intraoperative CT scans. The navigation transfer offered sufficiently accurate data to tailor the preprinted extended skull base defect prostheses. Successful implantation of the skull base prostheses was achieved in all specimens. The progressive flexibility gradient of the models’ edges offered the best compromise for easy intranasal maneuverability, anchoring, and structural stability. Prostheses printed based on intraprocedure CT scans were accurate in shape but slightly undersized. | 4 |
| 80 | Warren FM, Balachandran R, Fitzpatrick JM, Labadie RF. Percutaneous cochlear access using bone-mounted, customized drill guides: demonstration of concept in vitro. *Otol Neurotol.* 2007;28(3):325-329. | Observational-Dx | 0 | Percutaneous cochlear access can be performed using bone-mounted drill guides that are custom made on the basis of preintervention computed tomographic scans. | Eight cadaveric specimens were subjected to the study protocol. In seven of eight specimens, the drill bit trajectory was accurate; it passed from the lateral cortex to the lateral wall of the cochlea without compromise of any critical structures. In one specimen, the access to the middle ear was achieved, but the incus was hit by the drill. The average shortest distance +/- standard deviation from the edge of the drill bit to the boundaries of the facial recess was 0.78 +/- 0.56 mm (chorda tympani), 2.00 +/- 1.06 mm (incus buttress), and 1.27 +/- 0.54 mm (facial nerve). | 3 |
| 81 | Lopponen H, Holma T, Sorri M, et al. Computed tomography data based rapid prototyping model of the temporal bone before cochlear implant surgery. *Acta Otolaryngol Suppl.* 1997;529:47-49. | Review/Other- Dx | 0 | Rapid prototyping (RP) technique allows automatic fabrication of 3D model parts. This method was applied to make a temporal bone model before cochlear implant surgery. | In this prototype model the anatomy of the temporal bone can be clearly visualised, including, e.g., mastoid cells, tympanic cavity, bony canal of facial nerve, and round and oval windows. The inner ear spaces including vestibule, semicircular canals and cochlear turn are also shaped. | 4 |
| 82 | Mukherjee P, Cheng K, Flanagan S, Greenberg S. Utility of 3D printed temporal bones in pre-surgical planning for complex BoneBridge cases. *Eur Arch Otorhinolaryngol.* 2017;274(8):3021-3028. | Review/Other- Tx | 16 | In this study, 3D printed temporal bones of patients were used to study its utility in preoperative planning on complicated cases. | There was a statistically significant benefit in using 3D printed temporal bones to plan surgery for difficult cases of BoneBridge surgery compared to the current standard. | 4 |
| 83 | Canzi P, Marconi S, Manfrin M, et al. From CT scanning to 3D printing technology: a new method for the preoperative planning of a transcutaneous bone-conduction hearing device. *Acta Otorhinolaryngol Ital.* 2018;38(3):251-256. | Observational-Tx | 0 | The aim of the present study was to assess the feasibility and utility of 3D printing technology in surgical planning of a transcutaneous bone-conduction hearing device (Bonebridge®) (BB), focusing on the identification of the proper location and placement of the transducer. | The BB positioning was successfully performed on all human temporal bones, with no difficulties in finding the proper location of the transducer. | 3 |
| 84 | Suzuki R, Taniguchi N, Uchida F, et al. Transparent model of temporal bone and vestibulocochlear organ made by 3D printing. *Anat Sci Int.* 2018;93(1):154-159. | Review/Other- Dx | 0 | We report herein an attempt to produce a transparent three-dimensional-printed model of the human ear. | The three-dimensional relationships of the semicircular canals, spiral turns of the cochlea, and internal acoustic meatus were well recognizable from every direction through the transparent surface resin. | 4 |
| 85 | Kamrava B, Gerstenhaber JA, Amin M, Har-El YE, Roehm PC. Preliminary Model for the Design of a Custom Middle Ear Prosthesis. *Otol Neurotol.* 2017;38(6):839-845. | Observational-Dx | 0 | Custom prostheses could be used to recreate the ossicular chain and improve hearing. | Our measurements of cadaveric incudes corresponded well with those from the medical literature. These measurements were combined with anatomical information from micro-CT allowing identification of critical features of the incus, which remained constant. Other model features were modified to increase stability and facilitate synthesis, including broadening and thickening of the lenticular process and the incudomalleolar articulation. 3-D printed incudal replacements based on this model readily fit into a cadaveric temporal bone and successfully bridged the gap between malleus and incus. | 3 |
| 86 | Nomura Y, Tanaka T, Kobayashi H, Kimura Y, Soejima Y, Sawabe M. A 3-Dimensional Model of the Human Round Window Membrane. *Ann Otol Rhinol Laryngol.* 2019;128(6_suppl):103S-110S. | Review/Other- Dx | 0 | The round window membrane (RWM) is small in size, making it difficult to clarify its shape and structure. The authors examined a 40x magnified 3-dimensional model of the human RWM to clarify its morphologic aspects and characteristics. | The contour of this RWM model was approximately elliptic, with a saddle shape. When illuminated from the scala tympani side, the surface facing the fossula exhibited dark anterior and clear posterior portions. A borderline appeared where the 2 portions were bound along the short axis of the ellipse. This borderline was identified as the line of inflection. Collagen fibers were shown to run parallel to the borderline in the posterior portion but were fanned out in the anterior portion. | 4 |
| 87 | Kuru I, Maier H, Muller M, Lenarz T, Lueth TC. A 3D-printed functioning anatomical human middle ear model. *Hear Res.* 2016;340:204-213. | Observational-Dx | Not clearly stated | We have built an anatomically based and functional middle ear model to serve as a reproducible test environment. | Our experiments regarding the sound transmission showed that the model has a similar behavior to a human middle ear. The transfer function has a resonance frequency at around 1 kHz, the stapes' response is almost constant for frequencies below the resonance and a roll-off is observed above the resonance. The tympanometry results show that the compliance of the middle ear model is similar to the compliance of a healthy human middle ear. | 4 |
| 88 | Dhanasingh A, Dietz A, Jolly C, Roland P. Human Inner-ear Malformation Types Captured in 3D. *J Int Adv Otol.* 2019;15(1):77-82. | Observational-Dx | Not clearly stated | The objective of this study is to capture the human inner-ear malformation types in 3D by segmenting the inner-ear structures from clinical CT (computed tomography) and MR (magnetic resonance) image datasets. | We identified 2x normal anatomy (NA) cochlea, 1x enlarged vestibular aqueduct syndrome (EVAS), 3x incomplete partition (IP) type-I, 4x IP type-II, 3x IP type-III, 5x common cavity (CC) and 5x cochlear hypoplasia (CH). 3D segmented models along with the 3D printed models showed huge variation in size, shape and the anatomy among the image data-sets analyzed. Volumetric analysis showed that on average, volume of CC was above 150mm3, volume of CH fell below 80mm3, Volume of NA, EVAS and IP-I were all around 85-105mm3 whereas the volume of IP-II was around 50mm3. | 3 |
| 89 | Kozin ED, Remenschneider AK, Cheng S, Nakajima HH, Lee DJ. Three-Dimensional Printed Prosthesis for Repair of Superior Canal Dehiscence. *Otolaryngol Head Neck Surg.* 2015;153(4):616-619. | Review/Other- Dx | Not clearly stated | We aim to 3-dimensionally print customized prostheses to resurface or occlude bony SCD defects. | The prostheses occupied the superior semicircular canal defect, reflected in postrepair computed tomography scans. | 4 |
| 90 | Hirsch JD, Vincent RL, Eisenman DJ. Surgical reconstruction of the ossicular chain with custom 3D printed ossicular prosthesis. *3D Print Med.* 2017;3(1):7. | Review/Other- Dx | 0 | Custom 3D printing an individualized ossicular prosthesis would be a potential solution for the wide range of anatomic variation encountered in the pathological middle ear, and could decrease the rate of post-operative prosthesis displacement by increasing the likelihood of a proper fit, in addition to decreasing surgical time. | Each prosthesis had unique measurements. Each of the four surgeons was able to correctly match the prosthesis model to its intended temporal bone. The chances of this occurring randomly are 1:1296. | 4 |
| 91 | Gargiulo P, Arnadottir I, Gislason M, Edmunds K, Olafsson I. New Directions in 3D Medical Modeling: 3D-Printing Anatomy and Functions in Neurosurgical Planning. *J Healthc Eng.* 2017;2017:1439643. | Review/Other- Tx | 1 | This paper illustrates the feasibility and utility of combining cranial anatomy and brain function on the same 3D-printed model, as evidenced by a neurosurgical planning case study of a 29-year-old female patient with a low-grade frontal-lobe glioma. | This methodology highlights the potential for advanced neurosurgical preparation, which can begin before the patient enters the operation theatre. Moreover, the work presented here demonstrates the workflow developed at the National University Hospital of Iceland, Landspitali, focusing on the processes of anatomy segmentation, fiber tract extrapolation, MRI/CT registration, and 3D printing. | 4 |
| 92 | Makris DN, Pappas EP, Zoros E, et al. Characterization of a novel 3D printed patient specific phantom for quality assurance in cranial stereotactic radiosurgery applications. *Phys Med Biol.* 2019;64(10):105009. | Observational-Tx | Not clearly stated | This work presents and evaluates a novel methodology for patient-specific pre-treatment plan verification, utilizing 3D printing technology | Visual inspection of the fused CT images suggests excellent geometric similarity between phantom and patient, also confirmed using similarity indices. HUs and densities agreed within one standard deviation except for the skin (modeled as 'bone') and sinuses (water-filled). GI comparison between the calculated distributions resulted in passing rates better than 97% (1%/1 mm). DVHs and dose-volume metrics were also in satisfying agreement. In addition to serving as a feasibility proof-of-concept, experimental absolute film dosimetry verified the computational study results. GI passing rates were above 90%. Results of this work suggest that employing the presented methodology, patient-equivalent phantoms (except for the skin and sinuses areas) can be produced, enabling literally patient-specific pre-treatment plan verification in intracranial applications. | 3 |
| 93 | Damon A, Clifton W, Valero-Moreno F, Quinones-Hinojosa A. Cost-Effective Method for 3-Dimensional Printing Dynamic Multiobject and Patient-Specific Brain Tumor Models: Technical Note. *World Neurosurg.* 2020;140:173-179. | Review/Other- Dx | Not clearly stated | This work addresses a gap in the literature describing a cost-effective and time-efficient means of creating dynamic brain tumor 3D-printed models. | A step-by-step methodology and demonstration of the software manipulation techniques required for creating cost-effective, multidimensional brain tumor models for patient education and surgical planning are exhibited using a detailed written guide, images, and a video display. | 4 |
| 94 | Liu S, Wang H, Wang C, et al. Dosimetry verification of 3D-printed individual template based on CT-MRI fusion for radioactive (125)I seed implantation in recurrent high-grade gliomas. *J Contemp Brachytherapy.* 2019;11(3):235-242. | Observational-Tx | 16 | To verify the accuracy and efficacy of three-dimensional printing individual template (3D-PIT) with computed tomography-magnetic resonance imaging (CT-MRI) fusion for radioactive iodine-125 (125I) seed implantation in high-grade brain gliomas. | Sixteen treatment areas were reported in our study. Median gross tumor volume (preoperative) of patients was 64.2 cm3, median needle number was 8, and median number of implanted 125I seeds was 60. For postoperative plans, the median D90, V100, and V200 was 152.1 Gy, 96.8%, and 49.1%, respectively, and 151.7 Gy, 97.0%, and 48.9%, respectively, in preoperative plans. Comparing with the preplanned cases, the dose of the target volume was slightly higher; the high-dose area of the target volume was larger in postoperative cases, but the difference was not statistically significant (*p* > 0.05). Actual dose conformity of the target volume was greater than preplanned, and the difference was not statistically significant (*p* > 0.05). Local control was 81.25% and 75% at 3 and 6 months after implantation, respectively. No serious early toxicities were observed. | 2 |
| 95 | Mackle EC, Shapey J, Maneas E, et al. Patient-Specific Polyvinyl Alcohol Phantom Fabrication with Ultrasound and X-Ray Contrast for Brain Tumor Surgery Planning. *J Vis Exp.* 2020(161). | Review/Other- Tx | 0 | A phantom was developed using real patient data as input and 3D printing of molds to fabricate a patient-specific head phantom comprising the skull, brain and tumor with both ultrasound and X-ray contrast. | The phantom was successfully tested during a surgical simulation in a virtual operating room. | 4 |
| 96 | Grosch AS, Schroder T, Schroder T, Onken J, Picht T. Development and initial evaluation of a novel simulation model for comprehensive brain tumor surgery training. *Acta Neurochir (Wien).* 2020;162(8):1957-1965. | Review/Other- Tx | Not clearly stated | In this study, we introduce a novel simulator for realistic neurosurgical training that combines real brain tissue with 3D printing and augmented reality. | Visual and sensory realism of the skull and brain tissue were rated,"very good," while the sensory and visual realism of the tumor model were rated "good." Both overall satisfaction with the model and eligibility of the microscope and neurosurgical instruments for training purposes were rated with "very good." However, small size of the calf's brain, its limited shelf life, and the inability to simulate bleedings due to the lack of perfusion were significant drawbacks. | 4 |
| 97 | Waran V, Narayanan V, Karuppiah R, Owen SL, Aziz T. Utility of multimaterial 3D printers in creating models with pathological entities to enhance the training experience of neurosurgeons. *J Neurosurg.* 2014;120(2):489-492. | Review/Other- Tx | Not clearly stated | The authors used the latest generation of 3D printer to create a model, with an inbuilt pathological entity, of varying consistency and density. | sing this model the authors were able to take trainees through the basic steps, from navigation and planning of skin flap to performing initial steps in a craniotomy and simple tumor excision. | 4 |
| 98 | He X, Liu M, Zhang M, et al. A novel three-dimensional template combined with MR-guided (125)I brachytherapy for recurrent glioblastoma. *Radiat Oncol.* 2020;15(1):146. | Observational-Tx | 24 | At present, the treatment of recurrent glioblastoma is extremely challenging. In this study, we used a novel three-dimensional non-coplanar template (3DNPT) combined with open MR to guide 125I seed implantation for recurrent glioblastoma. The aim of this study was to evaluate the feasibility, accuracy, and effectiveness of this technique. | There were no significant differences between preoperative and postoperative dosimetry parameters of D90, V100, V200, CI, EI (P > 0.05). The ORR at 6 months was 75.0%. The 1-year survival rate was 58.3%. Median OS was 12.9 months. One case of small amount of epidural hemorrhage occurred during the procedure. There were 3 cases of symptomatic brain edema after brachytherapy treatment, including grade three toxicity in 1 case and grade two toxicity in 2 cases. The three patients were treated with corticosteroid for 2 to 4 weeks. The clinical symptoms related to brain edema were significantly alleviated thereafter. | 2 |
| 99 | Ploch CC, Mansi C, Jayamohan J, Kuhl E. Using 3D Printing to Create Personalized Brain Models for Neurosurgical Training and Preoperative Planning. *World Neurosurg.* 2016;90:668-674. | Observational-Dx | Not clearly stated | In this technical note, the authors present a new technology to create deformable, personalized models of the human brain. | In mechanical tests, the model stiffness (E = 25.29 ± 2.68 kPa) was 5 orders of magnitude softer than common 3D printed materials, and less than an order of magnitude stiffer than mammalian brain tissue (E = 2.64 ± 0.40 kPa). In a multicenter surgical survey, model size (100.00%), visual appearance (83.33%), and surgical anatomy (81.25%) were perceived as very realistic. The model was perceived as very useful for patient illustration (85.00%), teaching (94.44%), learning (100.00%), surgical training (95.00%), and preoperative planning (95.00%). | 3 |
| 100 | Lan Q, Zhu Q, Xu L, Xu T. Application of 3D-Printed Craniocerebral Model in Simulated Surgery for Complex Intracranial Lesions. *World Neurosurg.* 2020;134:e761-e770. | Review/Other- Tx | 49 | To demonstrate the use of 3-dimensional (3D)-printed intracranial lesion models for complex neurosurgery to increase the success rate of clinical surgeries via practice in simulated surgeries. | The 3D-printed brain tumor models were used to design the surgical route, to simulate piecemeal resection of tumors through keyhole approach, and to verify the extent of tumor resection. A drill was used for bone flap removal and milling of bony structures such as the anterior clinoid process, tuberculum sellae, petrous apex, and internal acoustic meatus. The tumors were removed by laser knife and cavitron ultrasonic aspiration. The 3D-printed aneurysm models were used to assess the feasibility of different keyhole approaches and to select the aneurysm clip. Actual surgery was based on the results of the simulated surgery. Postoperative MR image review showed that 84% (21/25) of patients had total tumor resection and 16% (4/25) subtotal resection. Digital subtraction angiography confirmed complete clipping of all aneurysms (24 cases/39 aneurysms). | 4 |
| 101 | van de Belt TH, Nijmeijer H, Grim D, et al. Patient-Specific Actual-Size Three-Dimensional Printed Models for Patient Education in Glioma Treatment: First Experiences. *World Neurosurg.* 2018;117:e99-e105. | Review/Other- Tx | 11 | We created patient-specific three-dimensional (3D) models of tumors including surrounding functional areas and assessed what patients with glioma value (or fear) about the models when they are used to educate them about the relationship between their tumor and specific brain parts, the surgical procedure, and risks. | Models were successfully created for all 11 participants. There were 18 facilitators and 8 barriers identified. The model improved patients' understanding about their situation; patients reported that it was easier to ask their neurosurgeon questions based on their model and that it supported their decision about preferred treatment. A perceived barrier for using the 3D model was that it could be emotionally confronting, particularly in an early phase of the disease. Positive effects were related to psychological domains, including coping, learning effects, and communication. | 4 |
| 102 | Brandmeir NJ, McInerney J, Zacharia BE. The use of custom 3D printed stereotactic frames for laser interstitial thermal ablation: technical note. *Neurosurg Focus.* 2016;41(4):E3. | Observational-Tx | 5 | The authors present the first report of a customized 3D printed stereotactic frame for LITT. | Intraoperative and postoperative imaging studies confirmed the accurate placement of the LITT catheter and the lesion created. Mean operating room time for all patients was 45 minutes but only 26 minutes when excluding the cases in which a biopsy was performed. | 3 |
| 103 | Thawani JP, Singh N, Pisapia JM, et al. Three-Dimensional Printed Modeling of Diffuse Low-Grade Gliomas and Associated White Matter Tract Anatomy. *Neurosurgery.* 2017;80(4):635-645. | Review/Other- Dx | 3 | To describe methods for rapid prototyping of DLGGs and surgically relevant anatomy. | This report represents a novel application of 3-dimensional (3-D) printing in neurosurgery and a means to model individualized tumors in 3-D space with respect to subcortical white matter tract anatomy. Faculty and resident evaluations of this technology were favorable at our institution. | 4 |
| 104 | Javan R, Davidson D, Javan A. Nerves of Steel: a Low-Cost Method for 3D Printing the Cranial Nerves. *J Digit Imaging.* 2017;30(5):576-583. | Review/Other- Dx | Not clearly stated | In this technical note, after manually perfecting the segmentation of each CN and brain stem on each SSFP-MRI image, initial 3D reconstruction was performed. | Two different methods are discussed for the key segmentation and 3D reconstruction steps, by either using professional commercial software, i.e., Materialise Mimics, or utilizing a combination of the widely available software Adobe Photoshop, as well as a freeware software, OsiriX Lite. | 4 |
| 105 | Bowen L, Benech R, Shafi A, et al. Custom-Made Three-Dimensional Models for Craniosynostosis. J Craniofac Surg. 2020;31(1):292-293. | Review/Other- Tx | 1 | The use of computer-aided manufacturing to improve the management of a case of nonsyndromic metopic synostosis was discussed. | A cutting guide for the cranium, custom-made orbital protectors, a 3D model of the predicted postoperative meninges to allow off the table bone recontouring, and a template frontal bar to allow more specific recontouring of the frontal bar were constructed | 4 |
| 106 | Soldozy S, Yagmurlu K, Akyeampong DK, et al. Three-dimensional printing and craniosynostosis surgery. Childs Nerv Syst. 2021;37(8):2487-2495. | Review/Other- Tx | 0 | The goal of this study was to review the current application and status of three-dimensional printing for craniosynostosis surgery. | A total of 15 studies were ultimately selected. This includes studies demonstrating novel three-dimensional simulation and printing workflows, studies utilizing three-dimensional printing for surgical simulation, as well as case reports describing prior experiences. | 4 |
| 107 | Andrew TW, Baylan J, Mittermiller PA, et al. Virtual Surgical Planning Decreases Operative Time for Isolated Single Suture and Multi-suture Craniosynostosis Repair. Plast Reconstr Surg Glob Open. 2018;6(12):e2038. | Observational-Tx | 66 | Data were retrospectively collected on patients who underwent craniosynostosis repair during a 7-year period. Information was collected on patient demographics, intraoperative and postoperative factors, and intraoperative surgical time. High-resolution computed tomography scans were used for preoperative planning with engineers when designing osteotomies, bone flaps, and final positioning guides. | A total of 66 patients underwent open craniosynostosis reconstruction between 2010 and 2017. There were 35 control (non-VSP) and 28 VSP cases. No difference in age, gender ratios, or number of prior operations was found. Blood loss was similar between the 2 groups. The VSP group had more screws and an increased length of postoperative hospital stay. The length of the operation was shorter in the VSP group for single suture and for multiple suture operations. Operative time decreased as the attending surgeon increased familiarity with the technique. | 2 |
| 108 | Dumas BM, Nava A, Law HZ, et al. Three-Dimensional Printing for Craniofacial Surgery: A Single Institution's 5-Year Experience. Cleft Palate Craniofac J. 2019;56(6):729-734. | Observational-Tx | 106 | To inform surgeons considering adoption of this evolving 3D printing technology, this study describes one multi-surgeon center's 5-year experience using a 3D printer | A total of 106 models were printed at this institution during the 5-year time period. Printing times were 7.4 ± 1.9 hours for complete skulls and 6.0 ± 1.7 hours for maxillofacial prints. The average cost for a complete skull was about US$60 in material cost alone. The 3D printer was most frequently used for complex craniosynostosis, hemifacial microsomia syndrome, and fibrous dysplasia cases. The surgeons found the printer to be most useful for planning complex facial orthognathic cases and least useful for routine single-suture synostosis. | 2 |
| 109 | Jimenez Ormabera B, Diez Valle R, Zaratiegui Fernandez J, Llorente Ortega M, Unamuno Inurritegui X, Tejada Solis S. [3D printing in neurosurgery: a specific model for patients with craniosynostosis]. Neurocirugia (Astur). 2017;28(6):260-265. | Observational-Tx | 4 | Acrylonitrile butadiene styrene plastic skull models were designed and printed from CT images of patients between 3 and 6 months of age with craniosynostosis of different sutures. The models were used to simulate surgical procedures. | Four models of four patients with craniosynostosis were produced: two with closure of the metopic suture and two with sagittal suture closure. The mean age of the patients was 5 months (3–6 m) and the mean duration of the surgery was 286 min (127–380 min). The acrylonitrile butadiene styrene plastic models printed for the project proved to be optimal for the simulation of craniosynostosis surgeries, both anatomically and in terms of mechanical properties and reaction to surgical instruments. | 4 |
| 110 | Elbanoby TM, Elbatawy AM, Aly GM, Sharafuddin MA, Abdelfattah UA. 3D printing guided surgery in the treatment of unicoronal craniosynostosis orbital dysmorphology. *Oral Maxillofac Surg.* 2020;24(4):423-429. | Observational-Tx | 16 | A retrospective analysis of 16 consecutive patients with unicoronal synostosis corrected by FOA guided by a guide model. | The study included nine males and seven females. The mean age of the patients at the time of the operation was 20.4 months. The mean follow-up duration was 36 months. Mean operative time was 170 min, mean anesthetic time was 230 min, mean blood loss was 50–80 ml, and the average hospital stay was 4.4 days. No relapse that required surgical correction was reported. There were improvements in the orbital indices and volume to be near equal to the normal side. Excellent to good results were obtained in all patients according to the Whitaker classification system. |  |
| 111 | Kim PS, Choi CH, Han IH, Lee JH, Choi HJ, Lee JI. Obtaining Informed Consent Using Patient Specific 3D Printing Cerebral Aneurysm Model. J Korean Neurosurg Soc. 2019;62(4):398-404. doi:10.3340/jkns.2019.0092. | Review/Other-Dx | 20 | We created patient-specific 3D printed aneurysm models as an educational and clinical tool for patients undergoing aneurysm clipping surgery. | The 3D printed models were successfully made, and they precisely replicated the actual intracranial aneurysm structure of the corresponding patients. The use of the 3D model was associated with a higher understanding and satisfaction of preoperative patient education and consultation. On a 5-point Likert scale, the average level of understanding was scored as 4.7 (range, 3.0-5.0) in group I. In group II, the average response was 2.5 (range, 2.0-3.0). | 4 |
| 112 | Acar T, Karakas AB, Ozer MA, Koc AM, Govsa F. Building Three-Dimensional Intracranial Aneurysm Models from 3D-TOF MRA: a Validation Study. J Digit Imaging. 2019;32(6):963-70. doi:10.1007/s10278-019-00256-6. | Review/Other-Dx | 32 | To create realistic three-dimensional (3D) vascular models from 3D time-of-flight magnetic resonance angiography (3D-TOF MRA) of an intracranial aneurysm (IA) | The mean maximum aneurysm diameter obtained from four MRA evaluations was 8.49 mm, whereas it was 8.83 mm according to the CT 3D PAM measurement. The Wilcoxon test revealed slightly larger mean CT 3D PAM diameters than the MRA measurements. The Spearman's correlation test yielded a positive correlation between MRA and CT lengths of 3D PAMs. | 3 |
| 113 | Nagassa RG, McMenamin PG, Adams JW, Quayle MR, Rosenfeld JV. Advanced 3D printed model of middle cerebral artery aneurysms for neurosurgery simulation. 3D Print Med. 2019;5(1):11. doi:10.1186/s41205-019-0048-9. | Review/Other-Dx | 0 | The present study aimed to replicate patient-derived cranial anatomy, pathology and human tissue properties relevant to cerebral aneurysm intervention through 3D printing and 3D print-driven casting techniques. | A patient-derived 3D aneurysm model was constructed for a MCA aneurysm. Multiple cerebral aneurysm models, patient-derived and CAD, were replicated as hollow high-fidelity models. The final assembled simulator integrated six anatomical components relevant to the treatment of cerebral aneurysms of the Circle of Willis in the left cerebral hemisphere. These included models of the cerebral vasculature, cranial nerves, brain, meninges, skull and skin. The cerebral circulation was modeled through the patient-derived vasculature within the brain model. Linear and volumetric measurements of specific physical modular components were repeated, averaged and compared to the original 3D meshes generated from the medical imaging data. Calculation of the concordance correlation coefficient (ρc: 90.2%-99.0%) and percentage difference (≤0.4%) confirmed the accuracy of the models. | 4 |
| 114 | Wang JL, Yuan ZG, Qian GL, Bao WQ, Jin GL. 3D printing of intracranial aneurysm based on intracranial digital subtraction angiography and its clinical application. Medicine (Baltimore). 2018;97(24):e11103. doi:10.1097/MD.0000000000011103. | Review/Other-Dx | 7 | The study aimed to develop simulation models including intracranial aneurysmal and parent vessel geometries, as well as vascular branches, through 3D printing technology. | Seven neurosurgical residents and 15 standardization training residents received their simulation model training and gave high assessments for the educational course with the follow-up qualitative questionnaire. | 4 |
| 115 | Wang L, Ye X, Hao Q, et al. Comparison of Two Three-Dimensional Printed Models of Complex Intracranial Aneurysms for Surgical Simulation. *World Neurosurg.* 2017;103:671-679. | Observational-Dx | 6 | To compare two 3-dimensional (3D) printed models of intracranial aneurysms for simulation and training for complex aneurysm surgery. | Both 3D aneurysm models were accurate: the diameter, width, and neck of the aneurysms in the models were not significantly different from the computed tomography angiography data (P > 0.05). Furthermore, the models were useful for selecting clips before surgery. The whole 3D model improved understanding of the surgical view more than the regional model did (P < 0.05); however, the clip application felt more realistic in the regional model (P < 0.05). The process time for making the whole model is shorter (P < 0.05) but more expensive (P < 0.05) compared with the regional model. | 3 |
| 116 | Wang L, Ye X, Hao Q, Ma L, Chen X, Wang H et al. Three-dimensional intracranial middle cerebral artery aneurysm models for aneurysm surgery and training. J Clin Neurosci. 2018;50:77-82. doi:10.1016/j.jocn.2018.01.074. | Review/Other-Dx | 8 | To develop a realistic model of middle cerebral artery (MCA) aneurysms using three-dimensional (3D) printing for surgical planning, research, and training of neurosurgical residents. | There was good agreement in the model aneurysm diameter, width, and neck and the CTA data, with no significant difference (p > 0.05) among the groups. The simulator was useful for choosing the clips to use before surgery. The average response to each of the survey questions was greater than 3.85 (range 3.0-5.0) on a five-point scale. The 3D printed MCA aneurysm models were accurate. | 4 |
| 117 | Ryan JR, Almefty KK, Nakaji P, Frakes DH. Cerebral Aneurysm Clipping Surgery Simulation Using Patient-Specific 3D Printing and Silicone Casting. World Neurosurg. 2016;88:175-81. doi:10.1016/j.wneu.2015.12.102. | Review/Other-Dx | 9 | The authors developed a hands-on, dimensionally accurate model for aneurysm clipping using patient-derived anatomic data and three-dimensional (3D) printing. Design of the model focused on reproducibility as well as adaptability to new patient geometry. | Through the novel manufacturing process, a patient-derived simulacrum was developed for neurovascular surgical simulation. A follow-up qualitative study suggests potential to enhance current educational programs; assessments support the efficacy of the simulacrum. | 4 |
| 118 | Kondo K, Nemoto M, Masuda H, et al. Anatomical Reproducibility of a Head Model Molded by a Three-dimensional Printer. *Neurol Med Chir (Tokyo).* 2015;55(7):592-598. | Observational-Dx | 22 | The objective of this study was to evaluate the anatomical reproducibility and accuracy of these models by comparison with the CTA images on a monitor. | The microsurgical anatomy and arteries were favorably reproduced, apart from a few minute regions, in the rapid prototyping models. No significant difference was noted in the measured lengths of the main arteries between the CTA image and rapid prototyping model, but errors were noted in their thickness (p < 0.001). A significant difference was also noted in the longitudinal diameter of the cerebral aneurysm (p < 0.01). Regarding the CTA image as the gold standard, reproducibility of the microsurgical anatomy of skull bone and main arteries was favorable in the rapid prototyping models prepared using a 3D printer | 3 |
| 119 | Javan R, Herrin D, Tangestanipoor A. Understanding Spatially Complex Segmental and Branch Anatomy Using 3D Printing: Liver, Lung, Prostate, Coronary Arteries, and Circle of Willis. *Acad Radiol.* 2016;23(9):1183-1189. | Review/Other-Dx | Not clearly stated | This report illustrates the steps in development of custom 3D models that enhance the understanding of complex anatomy. | Anatomic models of the liver, lungs, prostate, coronary arteries, and the Circle of Willis were created. These models have advantages that include customizable detail, relative low cost, full control of design focusing on subsegments, color-coding potential, and the utilization of cross-sectional imaging combined with graphic design. | 4 |
| 119 | Javan R, Herrin D, Tangestanipoor A. Understanding Spatially Complex Segmental and Branch Anatomy Using 3D Printing: Liver, Lung, Prostate, Coronary Arteries, and Circle of Willis. *Acad Radiol.* 2016;23(9):1183-1189. | Review/Other- Tx | Not clearly stated | We present a novel method of 3D printing a brain that allows for the simulation of placement of all types of intracranial electrodes. | The final model was light and durable and reflected accurate details of the surface anatomy and some deep structures. Additionally, standard surgical depth electrodes could be passed through the model to reach deep structures without damaging the model. | 4 |
| 120 | Andereggen L, Gralla J, Andres RH, et al. Stereolithographic models in the interdisciplinary planning of treatment for complex intracranial aneurysms. *Acta Neurochir (Wien).* 2016;158(9):1711-1720. | Review/Other-Tx | 6 | A 3D rapid prototyping (RP) technique based on multimodal imaging data was evaluated for use in planning of treatment for complex aneurysmal configurations. | In all cases, the model provided a comprehensive 3D representation of relevant anatomical structures and improved understanding of related vessels. Based on the 3D model, primary bypass surgery with subsequent reconstruction of the aneurysm was then considered advantageous in all but one patient after simulation of multiple approaches. | 4 |
| 121 | Nagesh SVS, Hinaman J, Sommer K, et al. A simulation platform using 3D printed neurovascular phantoms for clinical utility evaluation of new imaging technologies. *Proc SPIE Int Soc Opt Eng.* 2018;10578. | Review/Other-Tx | Not clearly stated | We present a platform to accurately simulate clinical views of neuro-endovascular interventions and devices. The neuro-endovascular interventional phantom has a 3D printed cerebrovasculature model derived from a patient CT angiogram and embedded inside a human skull providing bone attenuation. | The percentage difference between automatic exposure selection for the neuro-intervention phantom and the SK-150 phantom was under 10%. By changing 3D printed models, various patient diseased anatomies can be simulated accurately with the necessary x-ray attenuation. Using this platform various interventional procedures were performed using new imaging technologies such as a high-resolution x-ray fluoroscope and a dose-reduced region-of-interest attenuator and differential temporally filtered display for enhanced interventional imaging. | 4 |
| 122 | Kaneko N, Mashiko T, Namba K, Tateshima S, Watanabe E, Kawai K. A patient-specific intracranial aneurysm model with endothelial lining: a novel in vitro approach to bridge the gap between biology and flow dynamics. *J Neurointerv Surg.* 2018;10(3):306-309. | Observational-Dx | Not clearly stated | To develop an in vitro model for studying the biological effect of complex-flow stress on endothelial cells in three-dimensional (3D) patient-specific vascular geometry. | The CFD study showed low wall shear stress and circulating flow in the apex of the basilar tip aneurysm, with linear flow in the parent artery. Confocal imaging demonstrated that the inner surface of the vascular model was evenly covered with monolayer endothelial cells. After 24 h of flow circulation, endothelial cells in the parent artery exhibited a spindle shape and aligned with the flow direction. In contrast, endothelial cells in the aneurysmal apex were irregular in shape and size. | 3 |
| 123 | Bairamian D, Liu S, Eftekhar B. Virtual Reality Angiogram vs 3-Dimensional Printed Angiogram as an Educational tool-A Comparative Study. *Neurosurgery.* 2019;85(2):E343-E349. | Review/Other-Dx | Not clearly stated | To investigate and compare the practicality and potential of 3D printed and VR models in a neurosurgical education context. | VR angiogram outperformed 3D printed model in terms of resolution. It had statistically significant advantage in ability to zoom, resolution, ease of manipulation, model durability, and educational potential. VR angiogram had a higher questionnaire total score than 3D models. The 3D printed models had a statistically significant advantage in depth perception and ease of manipulation. The results were independent of trainee year level, sequence of the tests, or anatomy. | 4 |
| 124 | Leal A, Souza M, Nohama P. Additive Manufacturing of 3D Biomodels as Adjuvant in Intracranial Aneurysm Clipping. *Artif Organs.* 2019;43(1):E9-E15. | Review/Other-Tx | 8 | We propose a novel method that allows the generation of a 3D biomodel of the IA region under investigation using additive manufacturing technology (AM). The aim of this study is the creation of a flexible 3D physical model (elastomer) through the AM technique, in order to allow the clip selection prior to the surgery. | At the end of the study, all 3D IA biomodels were reproduced for microsurgical clipping selection and it was possible to predict the metal clip to be used in the surgery. | 4 |
| 125 | Chivukula VK, Levitt MR, Clark A, et al. Reconstructing patient-specific cerebral aneurysm vasculature for in vitro investigations and treatment efficacy assessments. *J Clin Neurosci.* 2019;61:153-159. | Review/Other-Dx | 6 | In this study, in vitro models were created from three-dimensional rotational angiography (3DRA) of six patients harboring intracranial aneurysms using a multi-step process involving 3D printing, index of refraction matching and silicone casting that renders the models transparent for flow visualization. | Optical transparency was verified by using an index of refraction matched working fluid that replicated the mechanical behavior of blood. Synchrotron imaging of vessel lumen, aneurysmal sac and endovascular devices was successfully obtained, and dimensional errors were found to be O(100 μm). | 4 |
| 126 | Liu Y, Gao Q, Du S, et al. Fabrication of cerebral aneurysm simulator with a desktop 3D printer. *Sci Rep.* 2017;7:44301. | Review/Other-Dx | Not clearly stated | We developed a novel cerebral aneurysm simulator which can be better represented the dynamic bulging process of cerebral aneurysm | The clinical blood flow and pulsation pressure similar to the human can be well simulated, which can be used to train the neurosurgical residents how to clip aneurysms more effectively. | 4 |
| 127 | Lan Q, Chen A, Zhang T, et al. Development of Three-Dimensional Printed Craniocerebral Models for Simulated Neurosurgery. *World Neurosurg.* 2016;91:434-442. | Review/Other-Dx | Not clearly stated | To use three-dimensional (3D) printed craniocerebral models to guide neurosurgery and design the best operative route preoperatively. | The 3D printed hollow aneurysm model was highly representative of what was observed during the surgery. The model had realistic texture and elasticity and was used for preoperative simulation of aneurysm clipping for clip selection, which was the same as was used during the surgery. The craniocerebral aneurysm model clearly showed the spatial relation between the aneurysm and surrounding tissues, which can be used to select the best surgical approach in the preoperative simulation, to evaluate the necessity of drilling the anterior clinoid process, and to determine the feasibility of using a contralateral approach. The craniocerebral tumor and anatomic model showed the spatial relation between tumor and intracranial vasculatures, tractus pyramidalis, and functional areas, which was helpful 1) when selecting the optimal surgical approach to avoid damage to brain function, 2) for learning the functional anatomy of the craniocerebral structure, and 3) for preoperative selection of surgical spaces in the sellar region. | 4 |
| 128 | Frolich AM, Spallek J, Brehmer L, et al. 3D Printing of Intracranial Aneurysms Using Fused Deposition Modeling Offers Highly Accurate Replications. *AJNR Am J Neuroradiol.* 2016;37(1):120-124. | Observational-Dx | 10 | We evaluated the method of fused deposition modeling for the production of aneurysm models replicating patient-specific anatomy. | Reproduction of hollow aneurysm models was technically feasible in 8 of 10 cases, with aneurysm sizes ranging from 41 to 2928 mm(3) (aneurysm diameter, 3-19 mm). A high level of anatomic accuracy was observed, with a mean Dice index of 93.6% ± 2.4%. Obstructions were encountered in vessel segments of <1 mm. | 3 |
| 129 | Anderson JR, Thompson WL, Alkattan AK, et al. Three-dimensional printing of anatomically accurate, patient specific intracranial aneurysm models. *J Neurointerv Surg.* 2016;8(5):517-520. | Observational-Dx | 10 | To develop and validate a method for creating realistic, patient specific replicas of cerebral aneurysms by means of fused deposition modeling. | 3D printed aneurysm models were created for all 10 subjects. Good agreement was seen between the models and the source anatomy. Aneurysm diameter measurements of the printed models and source images correlated well (r=0.999; p<0.001), with no statistically significant group difference (p=0.4) or observed bias. The SDs of the measurements were 0.5 mm and 0.2 mm for source images and 3D models, respectively. 3D printed models could be imaged with flow via MRI. | 3 |
| 130 | Xu Y, Tian W, Wei Z, et al. Microcatheter shaping using three-dimensional printed models for intracranial aneurysm coiling. *J Neurointerv Surg.* 2020;12(3):308-310. | Review/Other-Tx | 9 | The purpose of this study was to investigate the application of three-dimensional (3D) printing technology in microcatheter shaping. | Nine cases of microcatheter shaping were satisfactory and shaping the needle was not necessary; no rebound was observed. The microcatheter was placed in an ideal position, and the stent-assisted method was used in three cases of wide-neck aneurysm. There were no complications related to surgery. | 4 |
| 131 | Russ M, O'Hara R, Setlur Nagesh SV, et al. Treatment Planning for Image-Guided Neuro-Vascular Interventions Using Patient-Specific 3D Printed Phantoms. *Proc SPIE Int Soc Opt Eng.* 2015;9417. | Review/Other-Dx | Not clearly stated | In this study the optimal workflow to obtain such phantoms from 3D data for interventionist to practice on prior to an actual procedure was investigated. | Various Circle of Willis and cardiac arterial geometries were used. The phantoms were tested for ischemic stroke treatment, distal catheter navigation, aneurysm stenting and cardiac imaging under angiographic guidance. | 4 |
| 132 | Khan IS, Kelly PD, Singer RJ. Prototyping of cerebral vasculature physical models. *Surg Neurol Int.* 2014;5:11. | Review/Other-Dx | 1 | We describe the method to manufacture such a model and review some of its uses in the context of treatment planning, research, and surgical training. | The model constructed was shown to be a very accurate depiction of the aneurysm and its associated vasculature. It was found to be useful, among other things, for surgical training and as a patient education tool. | 4 |
| 133 | Namba K, Higaki A, Kaneko N, Mashiko T, Nemoto S, Watanabe E. Microcatheter Shaping for Intracranial Aneurysm Coiling Using the 3-Dimensional Printing Rapid Prototyping Technology: Preliminary Result in the First 10 Consecutive Cases. *World Neurosurg.* 2015;84(1):178-186. | Review/Other-Tx | 10 | We report a preliminary series of intracranial aneurysms treated with a microcatheter shape determined by the patient's anatomy and configuration of the aneurysm, which was fabricated with a 3D printer aneurysm model. | All of pre-planned microcatheters matched the vessel and aneurysm anatomy. Seven required no microguidewire assistance in catheterizing the aneurysm whereas 3 required guiding of a microguidewire. All of the microcatheters accurately aligned the long axis of the aneurysm. The pre-planned microcatheter shapes demonstrated stability in all except in 1 large aneurysm case. | 4 |
| 134 | Sullivan S, Aguilar-Salinas P, Santos R, Beier AD, Hanel RA. Three-dimensional printing and neuroendovascular simulation for the treatment of a pediatric intracranial aneurysm: case report. *J Neurosurg Pediatr.* 2018;22(6):672-677. | Review/Other-Tx | 1 | The authors present the case of an 8-year-old boy with a fusiform intracranial aneurysm and documented progressive growth. | The patient was successfully treated after the authors rehearsed the placement of a flow diverter using a patient-specific 3D-printed replicator system model. | 4 |
| 135 | Ishibashi T, Takao H, Suzuki T, et al. Tailor-made shaping of microcatheters using three-dimensional printed vessel models for endovascular coil embolization. *Comput Biol Med.* 2016;77:59-63. | Review/Other-Tx | 26 | Our aim was to introduce "tailor-made" microcatheter shapes for coil embolization using three-dimensional (3D) printed vessel models. | Twenty-six patients (27 aneurysms) were treated using a total of 48 microcatheters shaped while referring to the 3D printed vessel model. Of the 48 catheters, only 9 (19%) required modification of the initial shape due to inappropriate positioning of the catheter. Only 29% of the catheter placements required repositioning due to catheter kick back. There were no procedure-related complications, including aneurysm rupture. The responses from assistants to a questionnaire administered after the embolizations on the usefulness of the technique were favorable. | 4 |
| 136 | Mashiko T, Otani K, Kawano R, et al. Development of three-dimensional hollow elastic model for cerebral aneurysm clipping simulation enabling rapid and low cost prototyping. *World Neurosurg.* 2015;83(3):351-361. | Review/Other-Tx | 12 | In this article, we explain the hollow elastic model prototyping method and report on the effects of applying it to presurgical simulation and surgical training. | Simulations using the hollow elastic model were performed in 12 patients. In all patients, the clipping proceeded as scheduled. The surgeon's postoperative assessment was favorable in all cases. This method enables easy fabrication at low cost. | 4 |
| 137 | Mashiko T, Otani K, Kawano R, et al. Development of three-dimensional hollow elastic model for cerebral aneurysm clipping simulation enabling rapid and low cost prototyping. World Neurosurg. 2015;83(3):351-361. | Review/Other-Tx | 12 | In this article, we explain the hollow elastic model prototyping method and report on the effects of applying it to presurgical simulation and surgical training. | Simulations using the hollow elastic model were performed in 12 patients. In all patients, the clipping proceeded as scheduled. The surgeon's postoperative assessment was favorable in all cases. This method enables easy fabrication at low cost. | 4 |
| 138 | Kono K, Shintani A, Okada H, Terada T. Preoperative simulations of endovascular treatment for a cerebral aneurysm using a patient-specific vascular silicone model. *Neurol Med Chir (Tokyo).* 2013;53(5):347-351. | Review/Other-Tx | 1 | We report preoperative simulations of endovascular treatment for a case with an unruptured wide-neck aneurysm of the anterior communicating artery using a patient-specific silicone model. | Although this is a single case, we demonstrate that the simulations are feasible and helpful for designing a treatment strategy and safe manipulation of endovascular devices by experiencing their behavior before actual treatment. | 4 |
| 139 | Wurm G, Lehner M, Tomancok B, Kleiser R, Nussbaumer K. Cerebrovascular biomodeling for aneurysm surgery: simulation-based training by means of rapid prototyping technologies. *Surg Innov.* 2011;18(3):294-306. | Review/Other-Dx | Not clearly stated | The authors created the solid skull and the cerebral vessels in different materials to simulate the real aneurysm when clipped. | Precise plastic replicas of complex anatomical data provide intuitive tactile views that can be scrutinized from any perspective. Hollowed out vessel sections allow serial clipping efforts, evaluation of different clips, and clip positions. The models can be used for accurate prediction of vascular anatomy, for optimization of teaching surgical skills, for advanced procedural competency training, and for patient counseling. | 4 |
| 140 | Wurm G, Lehner M, Tomancok B, Kleiser R, Nussbaumer K. Cerebrovascular biomodeling for aneurysm surgery: simulation-based training by means of rapid prototyping technologies. *Surg Innov.* 2011;18(3):294-306. | Review/Other-Dx | 13 | The authors describe their experience with a patient series in which this relatively new visualization method was used in surgery for cerebral aneurysms. | A prospective comparison of SL biomodels with intraoperative findings proved that the biomodels replicated the anatomical structures precisely. | 4 |
| 141 | Karmonik C, Anderson JR, Elias S, et al. Four-Dimensional Phase Contrast Magnetic Resonance Imaging Protocol Optimization Using Patient-Specific 3-Dimensional Printed Replicas for In Vivo Imaging Before and After Flow Diverter Placement. *World Neurosurg.* 2017;105:775-782. | Observational-Dx | 2 | Hemodynamics in cerebral aneurysms are currently investigated toward clinical efficacy using nonstandardized computational simulation techniques. At the same time, flow patterns and velocities are accessible by 4-dimensional phase contrast magnetic resonance imaging (4D pcMRI). Complexity of protocol design and imaging duration has limited the use of this technique in clinical imaging. A new approach is presented to overcome these limitations. | In all cases, major flow patterns were visualized well; smaller aneurysms posed a challenge because of limited spatial resolution, whereas larger aneurysms contained regions of low velocity resulting in limited contrast in the flow-sensitive images. After PED placement, ordered aneurysmal flow was disrupted and intra-aneurysmal velocity was reduced on average by 24.5% (range, 12.9-31.5%). Exploratory statistical analysis yielded a positive significant correlation (P < 0.01) between changes in inflow velocity and posttreatment intra-aneurysmal flow velocity. | 3 |
| 142 | Tsang AC, Lai SS, Chung WC, et al. Blood flow in intracranial aneurysms treated with Pipeline embolization devices: computational simulation and verification with Doppler ultrasonography on phantom models. *Ultrasonography.* 2015;34(2):98-108. | Review/Other-Dx | 0 | The aim of this study was to validate a computational fluid dynamics (CFD) simulation of flow-diverter treatment through Doppler ultrasonography measurements in patient-specific models of intracranial bifurcation and side-wall aneurysms. | CFD simulations showed drastic flow reduction after flow-diverter treatment in both aneurysms. The mean volume flow rate decreased by 90% and 85% for the bifurcation aneurysm and the side-wall aneurysm, respectively. Velocity contour plots from computer simulations before and after flow diversion closely resembled the patterns obtained by color Doppler ultrasonography. | 4 |
| 143 | Knox K, Kerber CW, Singel SA, Bailey MJ, Imbesi SG. Rapid prototyping to create vascular replicas from CT scan data: making tools to teach, rehearse, and choose treatment strategies. *Catheter Cardiovasc Interv.* 2005;65(1):47-53. | Review/Other-Dx | 2 | Our goal was to develop and prove the accuracy of a system that would allow us to re-create live patient arterial pathology. | Comparison of the images made directly from the patient and from the replica showed that with each step, the relationships were maintained, remaining within 3% of the original, but some smoothing occurred in the final computer manipulation. | 4 |
| 144 | Ho WH, Tshimanga IJ, Ngoepe MN, Jermy MC, Geoghegan PH. Evaluation of a Desktop 3D Printed Rigid Refractive-Indexed-Matched Flow Phantom for PIV Measurements on Cerebral Aneurysms. *Cardiovasc Eng Technol.* 2020;11(1):14-23. | Review/Other-Dx | 0 | This study presents results of an investigation into the feasibility of fabrication of such models suitable for particle image velocimetry (PIV) using a common 3D printing Stereolithography process and photopolymer resin. | An idealised geometry of a cerebral aneurysm was printed to demonstrate its applicability for PIV experimentation. The material was shown to have a refractive index of 1.51, which can be refractive matched with a mixture of de-ionised water with ammonium thiocyanate (NH4SCN). The images were of a quality that after applying common PIV pre-processing techniques and a PIV cross-correlation algorithm, the results produced were consistent within the aneurysm when compared to previous studies. | 4 |
| 145 | Benet A, Plata-Bello J, Abla AA, Acevedo-Bolton G, Saloner D, Lawton MT. Implantation of 3D-Printed Patient-Specific Aneurysm Models into Cadaveric Specimens: A New Training Paradigm to Allow for Improvements in Cerebrovascular Surgery and Research. *Biomed Res Int.* 2015;2015:939387. | Review/Other-Dx | 2 | To evaluate the feasibility of implanting 3D-printed brain aneurysm model in human cadavers and to assess their utility in neurosurgical research, complex case management/planning, and operative training. | The 3D aneurysm models were successfully implanted to the cadaveric specimens' arterial circulation in all cases. The features of the neck in terms of flexibility and its relationship with other arterial branches allowed for the practice of surgical maneuvering characteristic to aneurysm clipping. Furthermore, the relationship of the aneurysm dome with the surrounding structures allowed for better understanding of the aneurysmal local mass effect. Noticeably, all of these observations were done in a realistic environment provided by our customized embalming model for neurosurgical simulation. | 4 |
| 146 | Karakas AB, Govsa F, Ozer MA, Eraslan C. 3D Brain Imaging in Vascular Segmentation of Cerebral Venous Sinuses. J Digit Imaging. 2019;32(2):314-21. doi:10.1007/s10278-018-0125-4. | Review/Other-Dx | Not clearly stated | Our purpose is to document those identified during routine 3D venography created through 3D models using two-dimensional axial images for teaching and learning neuroanatomy. | These models have various benefits such as the ability to adjust properties, to convert two-dimension images of the patient into three-dimension images, to have different color options, and to be economical. | 4 |
| 147 | Govsa F, Karakas AB, Ozer MA, Eraslan C. Development of Life-Size Patient-Specific 3D-Printed Dural Venous Models for Preoperative Planning. *World Neurosurg.* 2018;110:e141-e149. | Review/Other-Dx | Not clearly stated | Our purpose was to document the dural venous sinuses (DVS) and their variations identified during routine 3-dimensional (3D) venography created through 3D models for the teaching of complex cerebral anatomy. | Geometrical changes between the neighboring DVS could be easily manipulated and explored from different angles. Modeling helped to conduct the examination in detail with reference to geometrical features of DVS, degree of asymmetry, its extension, location, and presence of hypoplasia/atresia channels. Challenging DVS anatomy was exposed with models of adverse anatomical variations of the DVS network, including highly angulated, asymmetrical view, narrowed lumens, and hypoplasia and atresia structures. It assisted us in comprehending spatial anatomy configuration of life-like models. | 4 |
| 148 | Conti A, Pontoriero A, Iati G, et al. 3D-Printing of Arteriovenous Malformations for Radiosurgical Treatment: Pushing Anatomy Understanding to Real Boundaries. Cureus. 2016;8(4):e594. | Review/Other-Dx | Not clearly stated | We describe a technique to improve the understanding of the complex AVM angioarchitecture by 3D prototyping of individual lesions. | The time required for the contouring of the target lesion was shorter when the surgeons used the 3D-printed model of the AVM (p=0.001). The average volume contoured without the 3D model was 5.6 ± 3 mL whereas it was 5.2 ± 2.9 mL with the 3D-printed model (p=0.003). The 3D prototypes proved to be spatially reliable. Surgeons were absolutely confident or very confident in all cases that the volume contoured using the 3D-printed model was plausible and corresponded to the real boundaries of the lesion. The total cost for each case was 50 euros whereas the cost of the 3D printer was 1600 euros. | 3 |
| 149 | Dong M, Chen G, Li J, et al. Three-dimensional brain arteriovenous malformation models for clinical use and resident training. *Medicine (Baltimore).* 2018;97(3):e9516. | Observational-Dx | Not clearly stated | To fabricate three-dimensional (3D) models of brain arteriovenous malformation (bAVM) and report our experience with customized 3D printed models of patients with bAVM as an educational and clinical tool for patients, doctors, and surgical residents. | 3D printed bAVM models were successful made. By neurosurgeons' evaluation, the printed models precisely replicated the actual bAVM structure of the same patients (n = 7, 97% concordance, range 95%-99% with average of < 2 mm variation). The use of 3D models was associated shorter time for preoperative patient education and consultation, higher acceptable of the procedure for patients and relatives, shorter time between obtaining intraoperative DSA data and the start of endovascular treatment. Thirty surgical residents from residency programs tested the bAVM models and provided feedback on their resemblance to real bAVM structures and the usefulness of printed solid model as an educational tool. | 4 |
| 150 | Shah A, Jankharia B, Goel A. Three-dimensional model printing for surgery on arteriovenous malformations. *Neurol India.* 2017;65(6):1350-1354. | Review/Other-Dx and Tx | 6 | We present the advantages of using three-dimensional (3D) printed models as a preoperative investigational modality. | The model depicted the precise nature of the compactness and location of the nidus in relationship to the skull. It was possible to clearly delineate the course, size, and number of feeding vessels and draining veins. The model made identification of the normal and abnormal vessels easier and assisted in the preparation and conduct of surgery. The model was made to scale and was placed beside the surgeon during the operation. The limitation of current technology was that the exact differentiation of arteries and veins by color coding was not possible. | 4 |
| 151 | Thawani JP, Pisapia JM, Singh N, et al. Three-Dimensional Printed Modeling of an Arteriovenous Malformation Including Blood Flow. *World Neurosurg.* 2016;90:675-683 e672. | Review/Other-Dx | Not clearly stated | Using existing imaging data, we generated a patient's giant AVM to scale. | A series of 3-dimensional (3D) models were generated and blood flow dynamics were represented. Faculty and resident surveys were positive regarding the technology. | 4 |
| 152 | Kaneko N, Ullman H, Ali F, et al. In Vitro Modeling of Human Brain Arteriovenous Malformation for Endovascular Simulation and Flow Analysis. *World Neurosurg.* 2020;141:e873-e879. | Review/Other-Dx and Tx | 0 | The purpose of this study was to evaluate a newly developed in vitro AVM model for embolic material testing, preclinical training, and flow analysis. | The manufacture of 3D in vitro AVM models delivers a realistic representation of human nidus vasculature and complexity derived from patients. The injection of liquid embolic agents performed in the in vitro model successfully replicated real-life treatment conditions. The model simulated the plug and push technique before penetration of the liquid embolic material into the AVM nidus. The 4D flow MRI results were comparable to computational fluid dynamics analysis. | 4 |
| 154 | Ionita CN, Mokin M, Varble N, et al. Challenges and limitations of patient-specific vascular phantom fabrication using 3D Polyjet printing. *Proc SPIE Int Soc Opt Eng.* 2014;9038:90380M. | Observational-Dx | Not clearly stated | Additive manufacturing (3D printing) technology offers a great opportunity towards development of patient-specific vascular anatomic models, for medical device testing and physiological condition evaluation. However, the development process is not yet well established and there are various limitations depending on the printing materials, the technology and the printer resolution. | The accuracy of the printed models was very good: distance analysis showed average differences of 120 μm between the patient and the phantom reconstructed volume dimensions. Most errors were due to residual support material left in the lumen of the phantom | 3 |
| 155 | Costa PF, Albers HJ, Linssen JEA, et al. Mimicking arterial thrombosis in a 3D-printed microfluidic in vitro vascular model based on computed tomography angiography data. *Lab Chip.* 2017;17(16):2785-2792. | Observational-Dx | Not clearly stated | Here we present a method for fabricating microfluidic chips containing miniaturized vascular structures that closely mimic architectures found in both healthy and stenotic blood vessels | By applying computational fluid dynamics (CFD) modeling a correlation in terms of flow fields and local wall shear rate was found between the original and miniaturized artery. The walls of the microfluidic chips were coated with human umbilical vein endothelial cells (HUVECs) which formed a confluent monolayer as confirmed by confocal fluorescence microscopy. The endothelialised microfluidic devices, with healthy and stenotic geometries, were perfused with human whole blood with fluorescently labeled platelets at physiologically relevant shear rates. After 15 minutes of perfusion the healthy geometries showed no sign of thrombosis, while the stenotic geometries did induce thrombosis at and downstream of the stenotic area. Overall, the novel methodology reported here, overcomes important design limitations found in typical 2D wafer-based soft lithography microfabrication techniques and shows great potential for controlled studies of the role of 3D vessel geometries and blood flow patterns in arterial thrombosis. | 3 |
| 156 | O'Hara RP, Chand A, Vidiyala S, et al. Advanced 3D Mesh Manipulation in Stereolithographic Files and Post-Print Processing for the Manufacturing of Patient-Specific Vascular Flow Phantoms. Proc SPIE Int Soc Opt Eng. 2016;9789. | Review/Other-Dx | Not clearly stated | This research aims to present advanced mesh manipulation techniques of stereolithographic (STL) files segmented from medical imaging and post-print surface optimization to match physiological vascular flow resistance. | The first method allows outlet 3D mesh manipulations to merge superfluous vessels into a single junction, decreasing the number of flow outlets and making it feasible to include smaller vessels. Next we introduced Boolean operations to eliminate the need to manually merge mesh layers and eliminate errors of mesh self-intersections that previously occurred. Finally we optimize support addition to preserve the patient anatomical geometry. For post-print surface optimization, we investigated various solutions and methods to remove support material and smooth the inner vessel surface. Solutions of chloroform, alcohol and sodium hydroxide were used to process various phantoms and hydraulic resistance was measured and compared with values reported in literature. The newly mesh manipulation methods decrease the phantom design time by 30 - 80% and allow for rapid development of accurate vascular models. | 4 |
| 157 | Martinez-Galdamez M, Escartin J, Pabon B, et al. Optical coherence tomography: Translation from 3D-printed vascular models of the anterior cerebral circulation to the first human images of implanted surface modified flow diverters. *Interv Neuroradiol.* 2019;25(2):150-156. | Review/Other-Dx | Not clearly stated | In this report, we share our experience of using 3D-printed neurovascular anatomy models to simulate and test the navigability of a commercially available OCT system and to show the application of this device in a patient treated with the new generation of surface modified flow diverters. | Use of intermediate catheters in the 3D-printed replicas was associated with better navigation of the OCT catheters in favorable anatomies but did not help as much in unfavorable anatomies. OCT image analysis of a PCOM aneurysm treated with Pipeline Embolization Device Shield demonstrated areas of unsatisfactory apposition with no thrombus formation. | 4 |
| 158 | Maza G, VanKoevering KK, Yanez-Siller JC, et al. Surgical simulation of a catastrophic internal carotid artery injury: a laser-sintered model. *Int Forum Allergy Rhinol.* 2019;9(1):53-59. | Review/Other-Tx | Not clearly stated | In this study we evaluate the role of a simplified laser-sintered model combined with standardized training in improving the effectiveness of managing an ICA injury endoscopically. | At the end of the study, time to hemostasis was reduced from 105.49 seconds to 40.41 seconds (p < 0.001). The volume of blood loss was reduced from 690 to 272 mL (p < 0.001), and the confidence scores increased in 95.7% of participants, from an average of 3 up to 8. | 4 |
| 159 | Xu WH, Liu J, Li ML, Sun ZY, Chen J, Wu JH. 3D printing of intracranial artery stenosis based on the source images of magnetic resonance angiograph. *Ann Transl Med.* 2014;2(8):74. | Review/Other-Dx | Not clearly stated | Three dimensional (3D) printing techniques for brain diseases have not been widely studied. We attempted to 'print' the segments of intracranial arteries based on magnetic resonance imaging. | Seven responders marked "grade 1" to 3D printing results, while one marked "grade 4". Therefore, 87.5% of the clinicians considered the 3D printing were successful. | 4 |
| 160 | Reddy AS, Liu Y, Cockrum J, et al. Construction of a comprehensive endovascular test bed for research and device development in mechanical thrombectomy in stroke. *J Neurosurg.* 2020:1-8. | Review/Other-Dx and Tx | Not clearly stated | This paper presents an affordable, versatile, and realistic benchtop simulation model for stroke. | The test bed was highly versatile and allowed realistic, radiation-free mechanical thrombectomy for stroke due to large-vessel occlusion with rapid exchange of geometries and phantom types. Of the transparent cerebrovascular phantoms tested, the 3D-printed phantom was the easiest to manufacture, the glass model offered the best visibility of the interaction between embolus and thrombectomy device, and the flexible model most accurately mimicked the endovascular system during device navigation. None of the phantoms modeled branches smaller than 1 mm or perforating arteries, and none underwent realistic deformation or luminal collapse from device manipulation or vacuum. The hydraulic system created physiological flow rate and pressure leading to iatrogenic embolization during thrombectomy in all phantoms. Embolus analogs with known fabrication technique, structure, and tensile strength were introduced and consistently occluded the middle cerebral artery bifurcation under physiological flow, and their interaction with the device was accurately visualized. | 4 |
| 161 | Wang Q, Guo W, Liu Y, et al. Application of a 3D-Printed Navigation Mold in Puncture Drainage for Brainstem Hemorrhage. *J Surg Res.* 2020;245:99-106. | Review/Other-Tx | 7 | The present study aimed to evaluate whether the application of a three-dimensional (3D)-printed navigation mold achieved good outcomes in the surgical treatment of brainstem hemorrhage. | In all cases, the operation was completed successfully; no patient died or contracted an infection intraoperatively. The end of the puncture tube was located in the hematoma cavity in all cases. The deviation distance ranged from 2.5 to 7.2, and this distance gradually reduced with improvements in the technique. The hematoma drainage achieved satisfactory postoperative outcomes, with improvements in symptoms such as respiratory failure and hyperthermia. | 4 |
